# Oral Antihypertensives for Nonsevere Pregnancy Hypertension: Systematic Review, Network Meta- and Trial Sequential Analyses

**DOI:** 10.1161/HYPERTENSIONAHA.121.18415

**Published:** 2022-01-04

**Authors:** Jeffrey N. Bone, Akshdeep Sandhu, Edgardo D. Abalos, Asma Khalil, Joel Singer, Sarina Prasad, Shazmeen Omar, Marianne Vidler, Peter von Dadelszen, Laura A. Magee

**Affiliations:** Department of Obstetrics and Gynaecology, University of British Columbia (UBC), Canada (J.N.B., A.S., S.P., S.O., M.V.).; Centro Rosarino de Estudios Perinatales, Rosario, Argentina (E.D.A.).; Fetal Medicine Unit, Department of Obstetrics and Gynaecology, St George’s University Hospitals, NHS Foundation Trust, United Kingdom (A.K.).; Vascular Biology Research Centre, Molecular and Clinical Sciences Research Institute, St George’s University of London, United Kingdom (A.K.).; School of Population and Public Health, UBC, Canada (J.S.).; Department of Women and Children’s Health, King’s College London, United Kingdom (P.v.D., L.A.M.).

**Keywords:** blood pressure, morbidity, network meta-analysis, proteinuria, sample size

## Abstract

We aimed to address which antihypertensives are superior to placebo/no therapy or another antihypertensive for controlling nonsevere pregnancy hypertension and provide future sample size estimates for definitive evidence.

**Methods:**

Randomized trials of antihypertensives for nonsevere pregnancy hypertension were identified from online electronic databases, to February 28, 2021 (registration URL: https://www.crd.york.ac.uk/PROSPERO/; unique identifier: CRD42020188725). Our outcomes were severe hypertension, proteinuria/preeclampsia, fetal/newborn death, small-for-gestational age infants, preterm birth, and admission to neonatal care. A Bayesian random-effects model generated estimates of direct and indirect treatment comparisons. Trial sequential analysis informed future trials needed.

**Results:**

Of 1246 publications identified, 72 trials were included; 61 (6923 women) were informative. All commonly prescribed antihypertensives (labetalol, other β-blockers, methyldopa, calcium channel blockers, and mixed/multi-drug therapy) versus placebo/no therapy reduced the risk of severe hypertension by 30% to 70%. Labetalol decreased proteinuria/preeclampsia (odds ratio, 0.73 [95% credible interval, 0.54–0.99]) and fetal/newborn death (odds ratio, 0.54 [0.30–0.98]) compared with placebo/no therapy, and proteinuria/preeclampsia compared with methyldopa (odds ratio, 0.66 [0.44–0.99]) and calcium channel blockers (odds ratio, 0.63 [0.41–0.96]). No other differences were identified, but credible intervals were wide. Trial sequential analysis indicated that 2500 to 10 000 women/arm (severe hypertension or safety outcomes) to >15 000/arm (fetal/newborn death) would be required to provide definitive evidence.

**Conclusions:**

In summary, all commonly prescribed antihypertensives in pregnancy reduce the risk of severe hypertension, but labetalol may also decrease proteinuria/preeclampsia and fetal/newborn death. Evidence is lacking for many other safety outcomes. Prohibitive sample sizes are required for definitive evidence. Real-world data are needed to individualize care.

## Abstract 2

Supplemental Digital Content is available in the text.

Hypertensive disorders of pregnancy are a leading cause of maternal, fetal, and newborn mortality and morbidity, worldwide. As such, a large proportion of antenatal care is devoted to their detection.

It is established that antihypertensive therapy is better than placebo or no antihypertensive therapy (placebo/no therapy) at decreasing the risk of severe maternal hypertension.^1^ A strategy of blood pressure (BP) control in pregnancy with antihypertensive therapy reduces severe hypertension without adversely affecting fetal/newborn death or illness, fetal growth restriction, or preterm birth.^2^ Importantly, severe hypertension is associated with heightened maternal and fetal/newborn risk, similar to (and independent of) preeclampsia, making severe hypertension an outcome worthy of avoidance, and not just one worthy of detection and treatment.^3^ Many national and international clinical practice guidelines now advocate BP control in pregnancy^4,5^

The choice of antihypertensive therapy in pregnancy remains controversial. In previous direct comparisons, no antihypertensive has been shown to be superior to others,^1^ even among the most commonly recommended: labetalol, other β-blockers, nifedipine, and methyldopa. However, there is substantial uncertainty around point estimates of effect, due (at least in part) to the limited number of high-quality randomized controlled trials (RCTs) comparing drugs directly. However, recent advances in meta-analytical methods now enable use of indirect evidence, through network meta-analysis (NMA), an approach that pools data from multiple different treatments and their comparisons, and not just from pairs of treatment versus control options, thereby allowing one to compare the relative effectiveness of several treatments.^6,7^

Using a Bayesian NMA framework,^8^ we sought to address which antihypertensive agent(s) is(are) superior to placebo/no therapy or other antihypertensives for lowering BP in nonsevere hypertension in pregnancy, without increasing fetal/newborn complications; and where no firm conclusions for the main outcomes could be drawn, estimate the additional number of trial participants required to draw clinically relevant conclusions and guide clinical practice.

## Methods

This systematic review was prospectively registered and amendments documented (CRD42020188725) and did not require ethics approval as it involved completed research findings. The authors declare that all supporting data are available within the article and its Supplemental Materials.

Our search strategy of electronic databases, run from January 01, 2017 to February 28, 2021, mirrored that of the relevant Cochrane review run on September 13, 2017,^1^ without language restrictions (for details, see Table S1). Reference lists of retrieved studies were reviewed for additional eligible trials.

Included were all randomized trials of antihypertensives for nonsevere pregnancy hypertension (as the timeframe for treatment and place of care differ for severe hypertension), regardless of pregnancy hypertension type,^9^ previous antihypertensive treatment, or multiple gestation. Nonsevere hypertension was systolic BP 140 to 159 mm Hg and diastolic BP 90 to 109 mm Hg. Also, studies were included if women were described as having nonsevere or mild-moderate hypertension and a relevant BP range was specified.

Antihypertensive therapy was any pharmacological intervention to lower BP, regardless of route of administration or place of care. Treatment duration was ≥7 days. Comparators were placebo, no antihypertensive, or another antihypertensive, including agents of the same drug class and multi-drug approaches.

Trials were excluded if: they enrolled women postpartum, or >50% of women had severe hypertension at enrolment (ie, systolic BP ≥160 mm Hg or diastolic BP ≥110 mm Hg), unless women with nonsevere hypertension were reported separately; the intervention aimed to reduce preeclampsia risk (not BP); or there were unresolved data integrity concerns.

The main outcomes were severe hypertension, proteinuria/preeclampsia, fetal/neonatal death, small-for-gestational age (SGA) infants, preterm birth, and admission to neonatal care.

Secondary outcomes for the woman were need for additional antihypertensive (if BP goals were not achieved), changed/stopped drug due to maternal side effects, placental abruption, and Caesarean. Secondary outcomes for the baby were perinatal (fetal/newborn death, including miscarriage), respiratory distress syndrome (or respiratory support), and neonatal seizures.

The following core maternal outcomes in pregnancy hypertension were not included as they were reported by few trials or outcomes were too uncommon: maternal death, severe maternal morbidity, maternal admission to intensive care, intubation or ventilation other than for childbirth, and postpartum hemorrhage.^10^ We did not evaluate severe preeclampsia specifically, as it is variably defined,^9^ but used it when preeclampsia was not reported (for outcome definitions, see Table S2).

Search results were screened by 2 reviewers (S. Omar and S. Prasad) and full texts of all relevant reports retrieved and reviewed. Data were abstracted by 3 reviewers (Dr Abalos, S. Omar, and S. Prasad), using a review-specific form. Disagreement was resolved by consulting LA Magee and through consensus.

Included were all participants randomized who had known outcomes. Authors were contacted for clarification.

Quality was designated as low, high, or unclear based on: random sequence generation, allocation concealment, blinding of participants and personnel, blinding of outcome assessment, incomplete outcome data, and selective reporting.^11^ A trial was at high risk of bias overall if it were at high risk of bias in either random sequence generation or allocation concealment.

Our primary analysis focussed on antihypertensives consistently recommended in clinical guidelines and in common use: labetalol, β-blockers, methyldopa, calcium channel blockers (includes nifedipine), and a multi-drug group.

Where head-to-head trials were available, Bayesian meta-analysis was used compare each antihypertensive with placebo/no therapy or another antihypertensive. Results were summarized with odds ratios (ORs) and 95% credible intervals (CI). Heterogeneity was summarized using the *I*^*2*^ statistic and classified as: may not be important (*I*^*2*^<40%), may represent moderate heterogeneity (30%–60%), may represent substantial heterogeneity (50%–90%), and considerable (≥75%).^11^

For the NMA, a Bayesian random-effects model was used to generate estimates of direct and indirect treatment comparisons.^8^ For any 2 interventions, direct estimates were obtained by pooling data from head-to-head trials that compared those interventions, while indirect estimates were obtained by pooling data from trials through all common comparators. Trials with 3 arms had their data assigned to the relevant groups, so there was no double-counting. Rather than discarding trials (or applying continuity corrections) to studies with zero outcomes in at least one arm, the Bayesian approach naturally incorporates these as plausible values under the assumed prior distributions.

For each main outcome, we summarized network characteristics and created network plots to visualize direct and indirect paths, with the thickness of lines proportional to the number of available trials. Outcomes were summarized with league tables showing the combined network (direct+indirect) OR and 95% CI for each comparison.^12^

In both the meta-analysis and NMA, trace plots were used to assess convergence of the Markov Chain Monte Carlo algorithms; all models were fit on 5000 burn-in samples and 25 000 Markov Chain Monte Carlo iterations.^13^ Noninformative priors were used throughout.

Heterogeneity was quantified for each main outcome by an overall inconsistency index between direct and indirect ORs, the Bayesian NMA version of *I*^2^. *P*<0.05 was considered statistically significant.

Publication bias for main outcomes was assessed when there were ≥10 informative trials. Funnel plots were used to assess asymmetry visually and if asymmetry were suggested, an exploratory analysis was planned to investigate it via Egger test or the trim-and-fill method.

Sensitivity analyses were undertaken to (1) include trials of differential BP control; (2) include trials of antihypertensives less-commonly prescribed; and (3) exclude trials at high risk of bias. In analysis (1), data from tight BP control arms were assigned to multi-drug therapy if multiple agents were used; data from less tight arms were assigned to the placebo/no therapy group, as treatment was similar in that additional antihypertensive was administered if BP rose to unacceptable levels.

All meta-analyses were conducted using the Bayesmeta package^14^ and NMA conducted using the BUGSnet package in R.^15^ A 95% CI that excluded 1.0 was statistically significant.

Trial sequential analysis (TSA) was undertaken to inform NMA drug versus drug comparisons having at least one head-to-head trial. We estimated future sample sizes needed to detect differences in outcome, varying the relative risk reduction (RRR) between 10% (for noninferiority) and 30% (for superiority), and assuming a 5% significance level, 90% power, and outcome rates at the median in the antihypertensive arm of placebo/no therapy trials. For secondary outcomes, we estimated needed sample size based on median event rate and a 20% RRR. Analyses were conducted using open-access software.^16^

## Results

Our search identified N=1247 publications, from electronic databases (N=1168), the relevant Cochrane review^1^ (N=73), and other sources (N=6). Duplicate publications were removed (N=474). Further exclusions were made following title/abstract review (N=689 plus N=4 ongoing trials) and full-text review (N=8, four of which were designated as awaiting classification because further information was requested from the authors or a specific editorial caution had been issued).^17–25^ Seventy-two trials were included—N=61 trials from the prior Cochrane review^1^ and N=11 new trials, N=8 previously excluded from the Cochrane review, because they compared antihypertensives within the same drug class or N=3 because they were trials of differential BP control.^26–96^ See Appendix in the Supplemental Material for the Preferred Reporting Items for Systematic Reviews and Meta-Analyses flow diagram (Figure S1) and checklist (Tables S3 and S4).

### Trial Characteristics

Table 1 presents characteristics of the 61 trials (6923 women) that reported one or more outcomes in this review; for details, see Table S5. Eleven trials had no clinical outcomes, did not contribute to any analyses, and are not discussed further.^29,32,35,39,42,58,90–92,94,95^

**Table 1.**
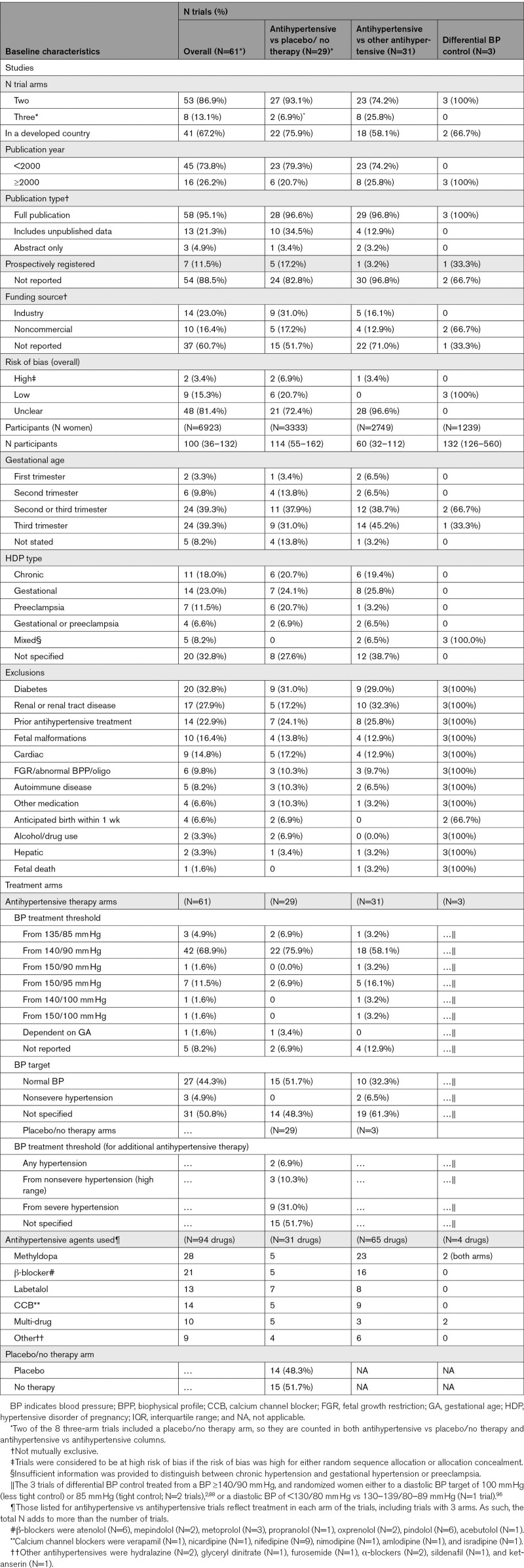
Baseline Characteristics of Participants and Interventions (N [%] or median [IQR] Unless Otherwise Specified)

Most trials had 2 treatment arms, were from developed countries, and were published before 2000, except for differential BP control trials that were all published thereafter. Most trials were detailed in full reports, but few were prospectively registered and most did not report their funding source. For the vast majority of trials, risk of bias was unclear; the 3 differential BP control trials were at low risk.

Participants numbered a median of 100, with antihypertensive versus antihypertensive trials at just under half that size. Most women were recruited in the second and particularly third trimesters, based most often on BP level, without specifying hypertensive disorder type. Exclusions were most commonly maternal, related to diabetes, renal disease, or prior antihypertensive treatment, whether the comparator was placebo/no therapy or another antihypertensive.

In the antihypertensive arms of trials (vs either placebo/no therapy or another antihypertensive), most trials initiated antihypertensives at a BP ≥140/90 mm Hg. While just under half of trials set a normal BP target (<140/90 mm Hg), half did not specify their BP goal. In placebo/no therapy arms, additional antihypertensive was often initiated when near-severe or severe hypertension developed, but most often (in half of trials), the criteria for additional antihypertensive were not stated. Most commonly, the antihypertensive studied was methyldopa (particularly in comparisons with another antihypertensive), followed by beta-blockers, and then calcium channel blockers and labetalol, and multi-drug therapy or other (ie, sildenafil, ketanserin, or glyceryl dinitrate). In placebo/no therapy trials, placebo and no therapy as comparators were equally common.

Antihypertensive versus placebo/no therapy and antihypertensive versus antihypertensive trials were similar. Trials of differential BP control were published later, at low risk of bias, and specified participants’ pregnancy hypertension type.

### Network Characteristics

For the most commonly used antihypertensives and each main outcome, the number of trials in each analysis varied from 16 to 34 (median 25), with 2818 to 4840 participants (median 3758). Trials had 2 treatment arms, except 3 trials that each had 3 arms. There were 15 pairwise comparisons possible for each outcome, with 12 to 15 direct pairwise comparisons possible, except for fetal/neonatal death for which only 9 were possible. Each outcome occurred in <20% of pregnancies, particularly for fetal/neonatal death (3.0%). Most studies had at least one event in each trial arm, with the exception of fetal/neonatal death, for which 14/23 trials lacked an event in one arm and 7/23 trials reported no event in any arm (for details of network characteristics, see Table S6).

For all outcomes, most information was from comparisons of placebo/no therapy with either labetalol (N=7 trials, 4448 women), methyldopa (N=5 trials, 2981 women), calcium channel blockers (N=5 trials, 4262 women), and beta-blockers (N=5 trials, 1342 women; Figure S2). Trials of methyldopa versus beta-blockers also contributed, particularly for the outcomes of severe hypertension and proteinuria. Trials with multi-drug arms in the network were only connected to the placebo/no therapy node. Networks were generally more sparsely populated with data for perinatal than maternal outcomes. Funnel plots showed no clear evidence of publication bias for any main outcomes (Figure S3).

Table 2 presents the results of the meta-analysis and NMA for the most commonly used antihypertensive medications and the 6 main outcomes. Meta-analysis revealed no evidence of substantial between-trial heterogeneity, based on *I*^2^<40% for most pairwise comparisons. In the NMA, there were few comparisons for which significant inconsistency was evident between direct and indirect evidence, and only for the outcome of severe hypertension; the magnitude of effect (rather than its direction) differed for each of methyldopa and calcium channel blockers versus placebo/no therapy. While the directions of effect for calcium channel blockers versus methyldopa were not consistent, the 95% CIs were wide, and there was no overall effect. There were too few trials within each relevant comparison to enable examination of moderation by trial-level covariates, as planned (ie, N=5 antihypertensive versus placebo/no therapy trials for each of methyldopa and calcium channel blockers, and N=5 calcium channel blocker versus methyldopa trials). The overall network results were consistent with the meta-analysis, but with narrower CIs.

**Table 2.**
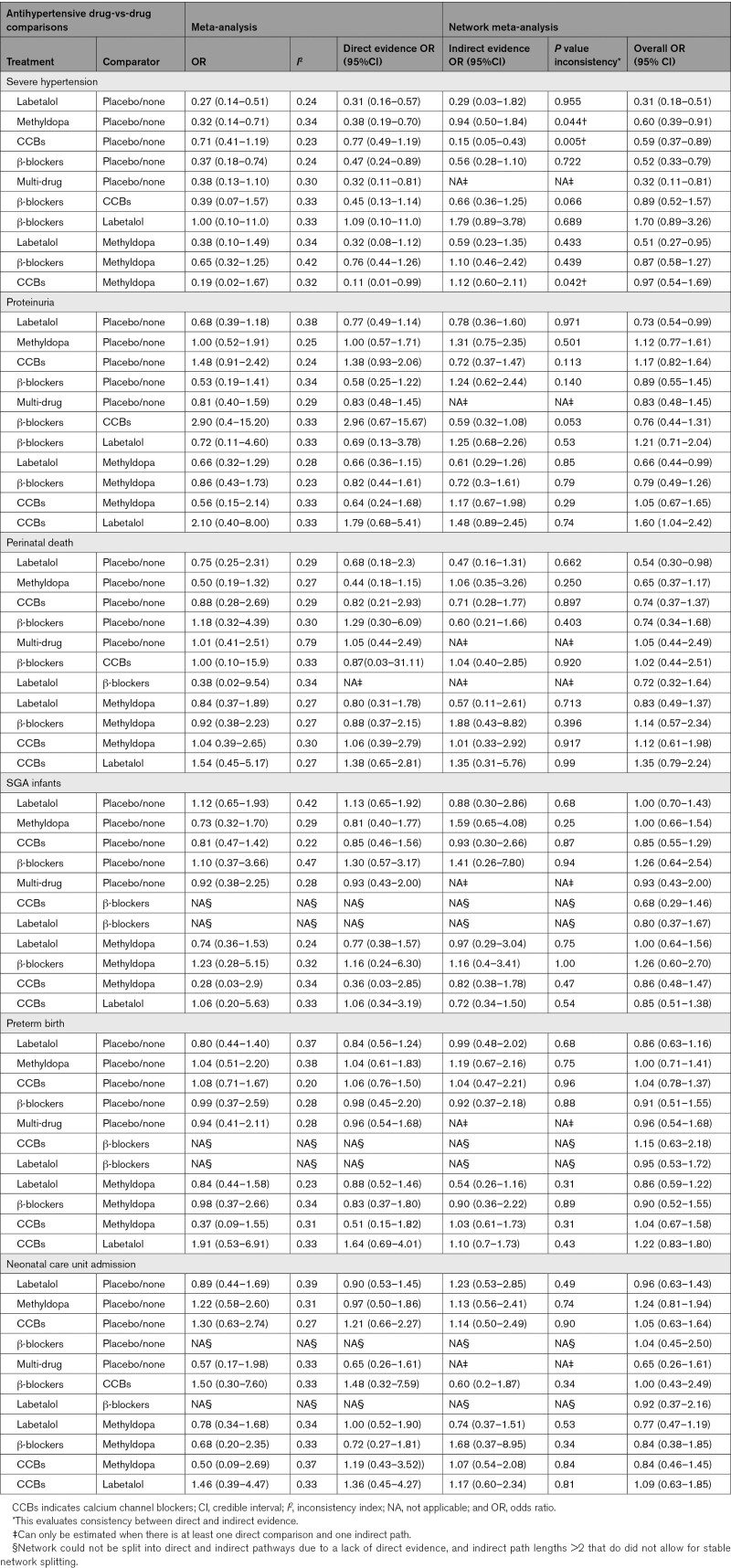
Antihypertensive Drug-vs-Drug Direct and Indirect Evidence, and Inconsistency Indices for the Main Outcomes*

**Figure 1.**
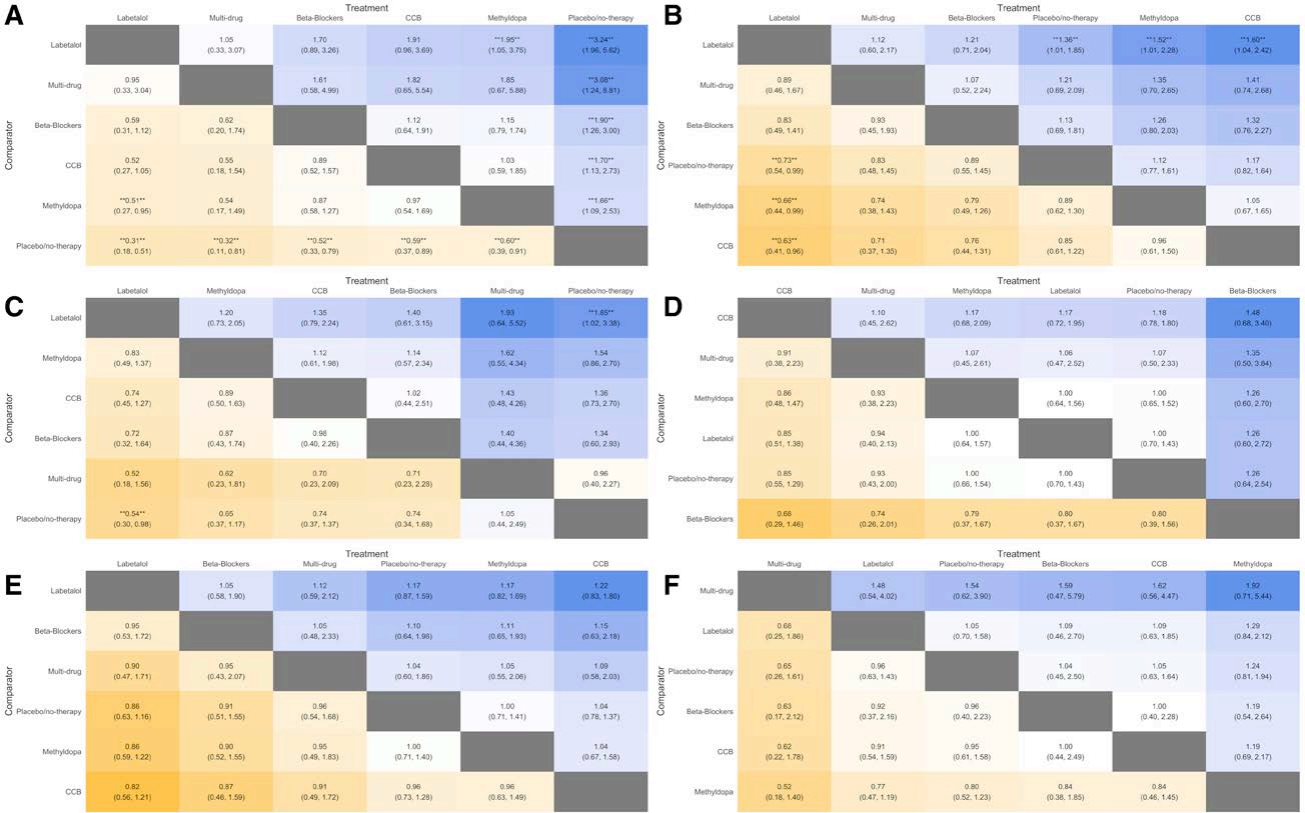
**League table comparing primary drugs of interest with placebo/no therapy and with each other.** All estimates are odds ratios (OR) and 95% credible intervals. Outcomes are (from **top left** to **bottom right**): severe hypertension (**A**), proteinuria (**B**), perinatal death (**C**), small-for-gestational age infants (**D**), preterm birth (**E**), and neonatal care unit admission (**F**). Blue represents an OR >1.0 and orange an OR <1.0, stronger effects are illustrated by darker colors, drugs with more favourable effects are located in columns to the **left**, and statistically significant results (at *P*<0.05) are marked by double asterisks.

The Figure 1 league table panels A and B show that for the mother, compared with placebo/no therapy, all of the most commonly used antihypertensives decreased the incidence of severe hypertension; effect estimates (shown along the bottom row) ranged from a 68% reduction in odds with multi-drug therapy (OR, 0.32 [95% CI, 0.12–0.80]) and 67% reduction with labetalol (OR, 0.33 [95% CI, 0.20–0.52]) on the left, to a 37% reduction with calcium channel blockers (OR, 0.63 [95% CI, 0.39–0.93]) on the right (Figure 1A). Compared with placebo/no therapy (shown in the fourth row), labetalol also decreased the incidence of proteinuria (OR, 0.73 [95% CI, 0.53–0.98]; Figure 1B); no significant effect was seen for other antihypertensives, but the point estimates were <1.0 for beta-blockers and multi-drug therapy, and >1.0 for methyldopa and calcium channel blockers. Also, labetalol decreased the incidence of severe hypertension compared with methyldopa (OR, 0.51 [95% CI, 0.27–0.95]), and proteinuria compared with either methyldopa (OR, 0.66 [95% CI, 0.44–0.99]) or calcium channel blockers (OR, 0.66 [95% CI, 0.41–0.96]). Otherwise, one antihypertensive drug was similar to another for mothers, but CIs were wide and consistent with up to a 5-fold difference in the odds of severe hypertension or a 2-fold difference in proteinuria.

The Figure 1 league table Panels C to F show that for the baby, labetalol decreased the incidence of perinatal death (OR, 0.54 [95% CI, 0.30–0.98]), with no other significant impact of antihypertensive therapy on outcomes, but CIs were wide, particularly for perinatal death (Figure 1C) and SGA infants (Figure 1D). Of note, the league table for SGA infants has labetalol and beta-blockers on the right, suggesting increased risk (with calcium channel blockers on the left), but for preterm birth, labetalol, and beta-blockers are on the left, suggesting decreased risk (with calcium channel blockers on the right; Figure 1E). There was no impact of any antihypertensive on neonatal care unit admission (Figure 1F).

### Secondary Outcomes

Many antihypertensives (vs placebo/no therapy) decreased the need for additional antihypertensive therapy (league table, Figure S4A; 23 trials, 2927 women): multi-drug therapy (OR, 0.29 [95% CI, 0.11–0.66]), labetalol (OR, 0.38 [95% CI, 0.21–0.66]), and calcium channel blockers (OR, 0.44 [95% CI, 0.22–0.88]); no significant effect was seen for methyldopa (OR, 0.66 [95% CI, 0.34–1.21]) or beta-blockers (0.65 [95% CI, 0.32–1.25]). Women were no more likely to change/stop drugs due to maternal side effects, but the 95% CIs were very wide (league table, Figure S4B; 20 trials, 1880 women). Antihypertensives (vs placebo/no therapy) had no impact on placental abruption (league table, Figure S4C; 5 trials, 1573 women) or Caesarean birth (league table, Figure S4D; 34 trials, 5877 women), or neonatal respiratory distress syndrome (Figure S4E; 7 trials, 1159 women), but there were extremely wide ranges of uncertainty for all but Caesarean. There were no significant differences between one antihypertensive and another for any of the secondary outcomes examined. Fetal or neonatal death (including miscarriage) and neonatal seizures could not be examined as outcomes as not enough studies reported them across the network (ie, N=7 and 3, respectively).

### Sensitivity Analyses

Results were not meaningfully altered following: (1) inclusion of the 3 trials of differential BP control^96,288^; (2) inclusion of trials evaluating antihypertensives less-commonly prescribed (eg, ketanserin); or (3) exclusion of the 2 trials at high risk of bias^40,44^ (Table S7).

#### Trial Sequential Analysis

**Figure 2.**
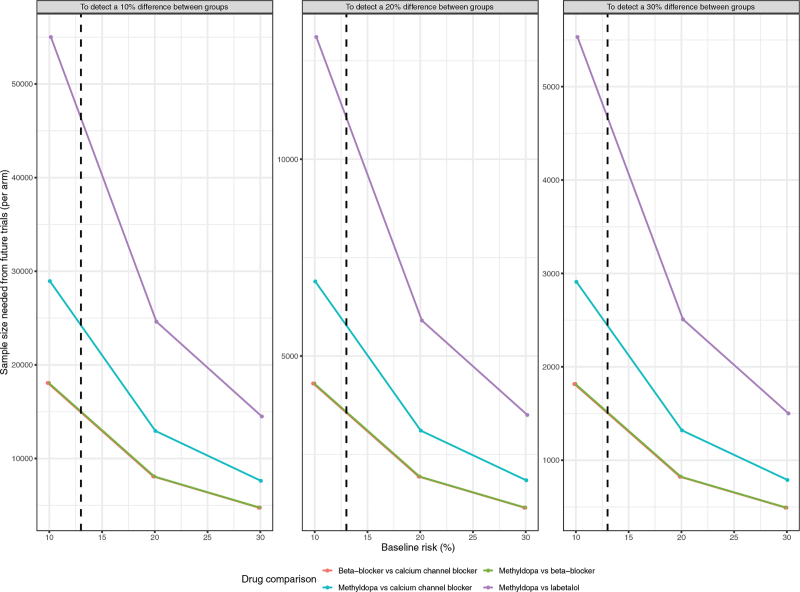
**Estimated sample size per arm from trial sequential analysis, for future drug-vs-drug to reach 5% statistical significance with 90% power for various risk reductions in severe hypertension.** The comparisons are β-blocker vs calcium channel blocker (red), methyldopa vs β-blocker (green), methyldopa vs calcium channel blocker (blue) and methyldopa vs labetalol (purple). The vertical dotted line represents a median severe hypertension rate of 13%.

Figure 2 shows TSA drug versus drug analyses for severe hypertension (median event rate of 13%, Table S3), of methyldopa versus labetalol (N=4, PURPLE), methyldopa versus calcium channel blockers (N=2, BLUE), methyldopa versus beta-blockers (N=5, GREEN), and β-blockers versus calcium channel blockers (N=1, RED). For superiority, the required sample size would be 2500 to 10 000/arm for a 20% RRR, varying from 12 500 to 45 000 for a 10% RRR to 400 to 1300/arm to 1250 to 4500/arm for a 30% RRR. Sample sizes were highest for methyldopa versus labetalol, and the lowest for β-blockers (not including labetalol) versus calcium channel blockers or methyldopa versus β-blockers. Data were not available for calculations for labetalol versus other β-blockers or calcium channel blockers (for which there is one ongoing trial of 1150 participants/arm).^18^ For the other main outcomes, sample sizes were similarly large for a 20% RRR, with particularly large samples required for perinatal death (ie, >15 000/arm; Table S8).

## Discussion

### Summary of Findings

By NMA of published RCTs of antihypertensive therapy for nonsevere hypertension in pregnancy, we have shown that compared with placebo/no therapy, antihypertensives used commonly, alone or in combination, reduce the odds of severe hypertension, by one- to two-thirds; similar reductions are seen for additional antihypertensive therapy. Labetalol is the only antihypertensive agent to decrease proteinuria and perinatal death, although 95% CI just exclude unity. There is no evidence of an impact of antihypertensives (vs placebo/no therapy) on other maternal outcomes (including Caesarean and placental abruption) or perinatal outcomes (including SGA, preterm birth, neonatal unit admission, and neonatal respiratory distress syndrome), although effect estimates were imprecise, particularly for less common outcomes, like fetal/newborn death.

Also, by NMA, there is no evidence that one antihypertensive is different from another with regards to reduction of severe hypertension risk, although labetalol decreases proteinuria compared with methyldopa or calcium channel blockers. The only evidence of differential impact of one drug versus another on perinatal outcomes is the reduction in perinatal mortality with labetalol.

While the 95% CI around estimates of antihypertensive drug versus drug effects are wide and consistent with important benefits or harms, our TSA showed that high future sample sizes would be needed to settle drug versus drug questions, even for the common outcome of severe hypertension. Most sample sizes for a realistic 20% reduction (for benefits) or increase (for harms) from median event rates observed, were often much higher than data either collected to date or anticipated from ongoing trials. No trial could be feasibly powered to examine fetal/newborn mortality.

### Interpretation

It is unsurprising that antihypertensive therapy outperforms, by a large margin, placebo/no therapy in lowering BP. Many national and international guidelines now recommend that practitioners offer antihypertensive therapy to women to normalize BP in pregnancy.^97^ Notwithstanding neonatal concerns, it may be reasonable to recommend first-line therapy with labetalol, given the additional potential benefits of reduced proteinuria/preeclampsia and perinatal death.^4^ Other reasonable antihypertensive choices include β-blockers, calcium channel blockers, labetalol, methyldopa, or multi-drug therapy, choices that make more likely that a hypertensive pregnant woman, regardless of the care setting, would have access to an antihypertensive drug.

Our findings are consistent with traditional meta-analysis that has refuted an association between β-blockers and fetal growth restriction.^1^ Notwithstanding, concerns about atenolol remain; this drug was not analyzed separately from β-blockers in this NMA, but was in another that examined antihypertensives for treatment of chronic hypertension in pregnancy, showing an association with SGA.^98^ There are many other beta-blockers available for use worldwide. Importantly, the pattern of contrary effects of antihypertensives on SGA and preterm birth, with no overall effect on neonatal morbidity (as measured by neonatal care unit admission), mirrors findings from the Control of Hypertension in Pregnancy Study trial.^99^

While pregnancy outcomes evaluated in RCTs are important, other considerations include drug availability, cost, drug dosage, and side effects (maternal and fetal/newborn). First, local drug availability can be a problem in any setting; labetalol is not licensed in many South American countries, and production of methyldopa or nifedipine has stopped in others. Second, while none of the antihypertensives used commonly in pregnancy is costly, the woman’s or system’s ability to pay may influence whether antihypertensive therapy is used at all. Third, when and how antihypertensives should be used in combination is not known; practice outside pregnancy suggests that it is more effective to administer lower doses of 2 or more drugs that act in different ways, rather than maximizing the dose of one drug first^100^ and this approach has also been used to optimize maternal hemodynamics in hypertensive pregnancy.^101^ Finally, potential side effects should influence drug choice. Although few women changed drugs (or withdrew) due to maternal side effects, the power to detect any between-group difference would be low, even in large RCTs. Many contraindications to antihypertensives are reasonably common, such as poorly controlled asthma (for which labetalol or β-blockers may exacerbate bronchospasm^102^), and depression (in which methyldopa is considered to increase the risk of postpartum depression^4^). Also, various pediatric societies recommend newborn monitoring for hypoglycemia following labetalol exposure, based on risks observed in observational studies.^103,104^

Given our findings and the prohibitively large sample sizes estimated by the TSA, even for the common outcome of severe hypertension, it is likely that we will continue to lack sufficient trial evidence to fully inform clinical care from maternal and fetal/newborn perspectives. However, real-world observational data (such as those from electronic health records) may provide the evidence necessary for individualized antihypertensive treatment that considers maternal phenotype (such as ethnicity and co-morbidity) and physiology (such cardiac output or heart rate). Advantages of using real-world data would include: evaluation of the impact of therapies on groups of women who are likely ineligible for trials (eg, hypertensive women with fetal growth restriction) or less likely to enrol in them (eg, ethnic minorities); assessment of antihypertensive impact on key but unusual outcomes, including some core outcomes in hypertensive pregnancy for mothers (eg, stroke) and babies (eg, seizures); and examination of long-term outcomes, for mothers (eg, cardiac remodeling and cardiovascular risk) and children (eg, neurodevelopment and metabolic outcomes related to developmental programming). Disadvantages include the greater potential for bias in real-world than RCT data. However, approaches have been developed to draw causal inference from routinely collected data, such as propensity scores and g-methods.^105^ These approaches are evolving,^106^ but have already led to important insights, such as the association between β-blockers and hypoglycemia, long-suspected but unproven in RCTs.^107^

### Strengths and Weaknesses

Strengths of our analysis include a comprehensive literature search to identify all relevant RCTs, including those of differential BP control not traditionally included. We considered labetalol (an α- and β-blocker) separately from β-blockers and the impact of trial quality on outcomes. We reported on a broad range of outcomes and considered the core outcomes for hypertensive pregnancy.^10^ Importantly, we used a Bayesian analytic approach that allows trials with zero events to inform the uncertainty around estimates. Finally, we used NMA, so our results are informed by both direct comparisons of interventions of interest and indirect evidence through drugs less-commonly used, thereby yielding indirect evidence for comparisons of interest for which there are few or no direct comparisons for all outcomes of interest (eg, labetalol versus nifedipine for severe hypertension).

Limitations include meta-analysis as a retrospective methodology constrained by the primary literature on which it is based. Many trials were >20 years old, so co-interventions (like hospitalization) may have been used differently, some details were missing (eg, trial registration), and it was unfeasible to obtain individual participant data that could inform treatment effects by baseline characteristics. There were too few trials in relevant comparisons to allow for a moderation analysis for trial-level covariates. We made many comparisons that risked finding significant results by chance, such as reductions in proteinuria and perinatal death with labetalol. There were core outcomes for hypertensive pregnancy for which too few trials reported the event (eg, neonatal seizures), or the trial event rates were too low for the network to be useful (eg, stroke); none of our main outcomes was reported by at least half of trials of which fewer reported perinatal outcomes. While we used up-to-date techniques for estimating heterogeneity in NMA, our models appeared to underestimate inconsistency between direct and indirect evidence; improved methods are required. In using a Bayesian approach for our meta-analysis and NMA and, therefore, the OR as our summary statistic, we were unable to compare our results directly with RRs from prior reviews^1^; however, OR may be better than RR when heterogeneity exists.^108^ Nevertheless, our CIs are slightly narrower than the Cochrane subgroup analyses, and estimates are more-or-less overlapping throughout. Finally, TSA could be undertaken only for some drug versus drug comparisons, given the need to have at least one direct comparison.

### Perspectives

The most commonly used antihypertensive agents in pregnancy all decrease the incidence of severe hypertension compared with placebo/no therapy, and labetalol also decreases proteinuria/preeclampsia, compared with placebo/no therapy, methyldopa, or calcium channel blockers. No other differences between antihypertensives were apparent, but 95% CIs were wide for important safety outcomes. Future trials would have to be prohibitively large. Taken together, these findings suggest that clinicians can individualize antihypertensive therapy in pregnancy by choosing from among those most commonly recommended until comprehensive information can be gathered from real-world care.

## Article Information

### Acknowledgments

We would like to thank the PRECISE (PREgnancy Care Integrating translational Science, Everywhere) team for their support. This is an honest, accurate, and transparent account of the study. No important aspects have been omitted. Changes from original plans have been explained (CRD42020188725). The prior relevant Cochrane review^1^ had PPI embedded. A James Lind Alliance Priority-Setting Partnership in pregnancy hypertension identified as a top-10 priority, how best to manage pregnancy hypertension with antihypertensives.^109^ Patients were not directly involved in the review, but results were interpreted in light of most women considering as important a range of pregnancy outcomes.

### Sources of Funding

UK Research and Innovation Grand Challenges Research Fund GROW Award (MRC/P027938/1). Dr Magee and von Dadelszen conceived the project and with J.N. Bone, designed the initial protocol, revised by all authors. Literature search and review were completed by S. Prasad and S. Omar. Analyses were by A. Sandhu and J.N. Bone. All authors reviewed results and interpretation, and revised the article for submission.

### Disclosures

None.

## Supplementary Material



## Novelty and Relevance

### What Is New?

All commonly used antihypertensive agents significantly decrease the risk of severe hypertension, but labetalol may also decrease the risk of proteinuria/preeclampsia and perinatal death.

### What Is Relevant?

All commonly used antihypertensive agents are not available in all care settings at all times.
Antihypertensive therapy of nonsevere hypertension in pregnancy is recommended by the International Society for the Study of Hypertension in Pregnancy, to a target diastolic blood pressure of 85 mm Hg, to decrease the risk of adverse maternal outcome, including severe hypertension.

### Clinical/Pathophysiological Implications?

Estimates of effect include important benefits and harms, but prohibitively large sample sizes would be required to provide definitive evidence for superiority of one of commonly used agent or class of agent over others. To provide timely evidence to inform clinical care, study designs are needed that facilitate evaluation of large numbers of participants with varied baseline characteristics, and the impact of relevant antihypertensive interventions on core outcomes.
