# Supplementary material for: Oral Antihypertensives for Nonsevere Pregnancy Hypertension: Systematic Review, Network Meta- and Trial Sequential Analyses
Source: Hypertension. 2022 Jan 4;79(3):614–28. doi: 10.1161/HYPERTENSIONAHA.121.18415 (PMC8823910; doi:10.1161/HYPERTENSIONAHA.121.18415)
Supplement: Supplementary file 1 [file hyp-79-614-s001.docx]

**SUPPLEMENTARY MATERIAL**

**Oral antihypertensives for non-severe pregnancy hypertension – systematic review, network meta- and trial sequential analyses**

Bone Jeffrey N, MSc^1^

Sandhu Ash, BSc^1^

Abalos Edgardo, DPhil^2^

Khalil Asma, MD^3^

Singer Joel, PhD^4^

Prasad Sarina, BSc^1^

Omar Shazmeen, BSc^1^

Vidler Marianne, PhD^1^

von Dadelszen Peter*, DPhil^4^

Magee Laura A*, MD^4^

** Shared senior authorship*

1. Department of Obstetrics and Gynaecology, University of British Columbia, Canada;
2. Centro Rosarino de Estudios Perinatales (CREP), Rosario, Argentina;
3. Fetal Medicine Unit, Department of Obstetrics and Gynaecology, St George’s University Hospitals, NHS Foundation Trust, UK;
4. School of Population and Public Health, University of British Columbia, Canada; and
5. Department of Women and Children’s Health, King’s College London, UK.

**TABLE OF CONTENTS**

|  | **Title** | **Page #** |
| --- | --- | --- |
| **Tables** | |  |
| Table S1 | Search strategy | 3 |
| Table S2 | Outcome definitions | 4 |
| Table S3 | Preferred Reporting Items for Systematic Reviews and Meta-Analyses (PRISMA) 2020 checklist | 6 |
| Table S4 | PRISMA 2020 checklist for abstracts | 9 |
| Table S5 | Characteristics of included trials | 10 |
| Table S6 | Characteristics of included trials by main outcomes for primary analysis | 22 |
| Table S7 | Sensitivity analyses of main outcomes for antihypertensives vs. placebo/no therapy, in comparison with results from the primary analysis | 23 |
| Table S8 | Trial sequential analyses sample sizes for detection of a relative risk reduction of 20% | 24 |
| **Figures** | |  |
| Figure S1 | PRISMA 2020 flow diagram | 25 |
| Figure S2 | Network plots for main outcomes | 26 |
| Figure S3 | Funnel plots for primary outcomes | 27 |
| Figure S4 | League table of secondary outcomes | 28 |

**Table S1**: Search strategy of electronic databases*

*CINAHL (The Cumulative Index to Nursing and Allied Health Literature), CENTRAL (Cochrane Central Register of Controlled Trials), ICTRP (International Clinical Trials Registry Platform), LILACS (Latin American and Caribbean Health Sciences Literature), WHO (World Health Organization)*

** The search strategy was run from 01 Jan 2017 to 28 Feb 2021, without language restrictions. Study dates referred to the latest for either publication or registration, on ClinicalTrials.gov or the WHO International Clinical Trials Registry Platform (ICTRP).*

| **Databases** | **Key words** |
| --- | --- |
| PubMed, Medline, Embase, CINAHL, CENTRAL, and Web of Science | |
|  | Filter: ‘Humans’  {hypertension, hypertensive disorders of pregnancy, pregnancy-induced hypertension, preeclampsia, pregnancy toxemias, OR gestational hypertension} AND  {antihypertensives OR antihypertensive agent} AND  {Controlled trial [pt] OR controlled clinical trial [pt] OR clinical trial [pt] OR randomized controlled trials [mh] OR random allocation [mh] OR double-blind method [mh] OR single-blind method [mh] OR (“clinical trial” [tw]) OR ((single*[tw] OR doubl*[tw] OR trebl*[tw] OR tripl*[tw]) AND (mask*[tw] OR blind*[tw])) OR placebos[tw] OR randomi*[tw] OR research design[mh:noexp] OR comparative study[pt] OR Evaluation Studies[PT] OR Evaluation Studies as Topic[mh] OR follow-up studies[mh] OR prospective studies[mh] OR control[tw] OR control[tw] OR controls[tw] OR controll* OR prospective*[tw] OR volunteer*[tw]} AND  {Pregnancy [mh] OR Pregnan* OR Gestation* OR pregnant women[mh] OR Pregnancy Complications[mh] OR “Postpartum Period”[Mesh] OR Puerperium OR postpartum OR “Peripartum Period”[Mesh] OR Peripartum* OR Perinatal Care[mh] OR perinatal} |
| **ClinicalTrials.gov** (advanced search) | |
|  | Hypertension, pregnancy induced (in Condition) AND intervention studies |
|  | Preeclampsia (in condition) AND intervention studies |
| **WHO ICTRP** | |
|  | Hypertension AND pregnancy |
|  | Preeclampsia AND pregnancy |
|  | Preeclampsia AND pregnancy |
| **LILACS and Cochrane Pregnancy & Childbirth Trials Register** | |
|  | {hypertension, hypertensive disorders of pregnancy, pregnancy-induced hypertension, preeclampsia, pregnancy toxemias, OR gestational hypertension} AND  {antihypertensives OR antihypertensive agent} |

**Table S2**: Outcome definitions

*BP (blood pressure), HELLP (haemolysis, elevated liver enzymes, low platelet), SGA (small-for-gestational age),*

** These are core outcomes in preeclampsia and defined as recommended (10). Gestational age is reported according to preterm birth, and birthweight as SGA infants.*

| **Outcome** | **Definition** |
| --- | --- |
| Primary |  |
| Severe hypertension | Whenever possible as systolic BP ≥160mmHg or diastolic BP ≥110 mmHg, but otherwise accepted as being up to 10 mmHg higher |
| Proteinuria/ preeclampsia | Whenever possible as new proteinuria (≥1+ by dipstick, ≥30mg/mmol protein:creatinine ratio, or ≥300 mg/24 hours |
| Fetal or newborn death* (including miscarriage) | Miscarriage was fetal loss before viability, usually taken as 20 or 24 weeks |
|  | Stillbirth was fetal death at ≥22+0 weeks, birthweight ≥500g, or crown-heel length ≥25cm. If this definition could not be applied, stillbirth was defined as reported |
|  | Neonatal death was newborn death in the first 28 days |
|  | Perinatal deaths were stillbirths plus neonatal deaths in the first week of life |
| SGA infants* | Birthweight < 10^th^ centile for gestational age, reported for all births (including stillbirths), and as assessed against a validated global, regional, or local customised growth chart. If this is not possible, alternative definitions will be accepted, including birthweight <3^rd^ centile for gestational age, and ‘low birthweight’, using the definition reported |
| Preterm birth* | Births before 37 weeks’ gestation |
| Neonatal unit admission* | As meeting the local, regional, or national criteria for admission to the special care baby unit or neonatal care unit |
| **Secondary (mother)** |  |
| Need for additional antihypertensive medication | As stated for both groups if BP goals were not achieved |
| Maternal death* | Death of the mother during pregnancy or within 6 weeks after birth |
| Eclampsia* | Onset of convulsions (i.e., fits, generalised convulsatons, tonic-clonic seizure or seizure) in a woman with not attributable to other causes |
| HELLP syndrome* | Low platelets* is a reduction in preeclampsia the number of platelets in the blood to <100,000/mL |
|  | Elevated liver enzymes* is an AST or ALT greater than at least twice the upper limit of normal |
| Severe maternal morbidity* |  |
| Stroke | In high-income countries, acute symptoms of focal brain injury lasting >24hr, with ischaemic or haemorrhagic stroke confirmed by neuroimaging  In low-income countries, acute symptoms of focal brain injury lasting >24hr |
| Cortical blindness | Visual impairment in presence of an intact pupillary response to light |
| Retinal detachment | A condition in which the retina peels away from its underlying layer of support tissue diagnosed by ophthalmological exam |
| Pulmonary edema | Clinical diagnosis of excess fluid in the lungs with chest x-ray confirmation or requirement of directive treatment and an oxygen saturation <95% |
| Acute kidney injury | Fulfills any of the following criteria:  (i) rise in serum creatinine ≥26μmol/L within 48hr  (ii) >50% rise in serum creatinine within the past 7d  (iii) urine output <0.5ml/kg/hr for >6hr  (iv) serum creatinine >150μmol/L in absence of baseline serum creatinine |
| Liver capsule hematoma or rupture | Blood collection under the hepatic capsule as confirmed by ultrasound, computerised tomography, magnetic resonance imaging, or laparotomy |
| Admission to intensive care* | Requirement for advanced respiratory support alone or monitoring and support for ≥2 organ systems |
| Intubation or ventilation* | Need for continuous positive airway pressure, non-invasive positive pressure ventilation, and intubation and mechanical ventilation |
| Placental abruption* | In the absence of placental previa on ultrasound, vaginal bleeding in the second or third trimester with either uterine irritability or labour or clinical signs and hypovolaemic shock or coagulopathy or placental pathology with histological findings of a chronic abruption |
| Antenatal hospital admission | Admission to hospital before the admission for birth |
| Antenatal hospital length of stay (LOS) >7 days | Total days in hospital for birth > 7 days |
| Cesarean | Surgical incision of the uterus to allow for birth of the baby |
| Changed/stopped drug due to maternal side-effects | The need to discontinue the allocated treatment because of maternal side-effects |
| Postpartum haemorrhage* | Perceived abnormal bleeding following delivery and hypotension and/or medical or surgical interventions for postpartum haemorrhage |
| **Secondary (baby)** |  |
| Respiratory distress syndrome (or respiratory support)* and | Need for continuous positive airway pressure, non-invasive positive pressure ventilation, and intubation and mechanical ventilation. If respiratory distress syndrome were specifically diagnosed, by clinical and/or radiological findings, this was included. |
| Neonatal seizures | In high-income countries, clinical recognition of neonatal seizures confirmed by electroencephalogram monitoring  In low-income countries, clinical recognition of neonatal seizures |

**Table S3:** Preferred Reporting Items for Systematic Reviews and Meta-Analyses (PRISMA) 2020 checklist

| **Section and Topic** | **Item #** | **Checklist item** | **Location item**  **is reported** |
| --- | --- | --- | --- |
| **TITLE** | | |  |
| Title | 1 | Identify the report as a systematic review. | 1 |
| **ABSTRACT** | | |  |
| Abstract | 2 | See the PRISMA 2020 for Abstracts checklist. | 3 |
| **INTRODUCTION** | | |  |
| Rationale | 3 | Describe the rationale for the review in the context of existing knowledge. | 4 |
| Objectives | 4 | Provide an explicit statement of the objective(s) or question(s) the review addresses. | 4 |
| **METHODS** | | |  |
| Eligibility criteria | 5 | Specify the inclusion and exclusion criteria for the review and how studies were grouped for syntheses. | 5 |
| Information sources | 6 | Specify all databases, registers, websites, organisations, reference lists and other sources searched or consulted to identify studies. Specify the date when each source was last searched or consulted. | 5 |
| Search strategy | 7 | Present the full search strategies for all databases, registers and websites, including any filters and limits used. | 5, Table S1 |
| Selection process | 8 | Specify the methods used to decide whether a study met the inclusion criteria of the review, including how many reviewers screened each record and each report retrieved, whether they worked independently, and if applicable, details of automation tools used in the process. | 5,6 |
| Data collection process | 9 | Specify the methods used to collect data from reports, including how many reviewers collected data from each report, whether they worked independently, any processes for obtaining or confirming data from study investigators, and if applicable, details of automation tools used in the process. | 6 |
| Data items | 10a | List and define all outcomes for which data were sought. Specify whether all results that were compatible with each outcome domain in each study were sought (e.g. for all measures, time points, analyses), and if not, the methods used to decide which results to collect. | 5,6 |
|  | 10b | List and define all other variables for which data were sought (e.g. participant and intervention characteristics, funding sources). Describe any assumptions made about any missing or unclear information. | 6 |
| Study risk of bias assessment | 11 | Specify the methods used to assess risk of bias in the included studies, including details of the tool(s) used, how many reviewers assessed each study and whether they worked independently, and if applicable, details of automation tools used in the process. | 6 |
| Effect measures | 12 | Specify for each outcome the effect measure(s) (e.g. risk ratio, mean difference) used in the synthesis or presentation of results. | 7 |
| Synthesis methods | 13a | Describe the processes used to decide which studies were eligible for each synthesis (e.g. tabulating the study intervention characteristics and comparing against the planned groups for each synthesis (item #5)). | 6,7 |
|  | 13b | Describe any methods required to prepare the data for presentation or synthesis, such as handling of missing summary statistics, or data conversions. | 6,7 |
|  | 13c | Describe any methods used to tabulate or visually display results of individual studies and syntheses. | 6,7 |
|  | 13d | Describe any methods used to synthesize results and provide a rationale for the choice(s). If meta-analysis was performed, describe the model(s), method(s) to identify the presence and extent of statistical heterogeneity, and software package(s) used. | 6,7 |
|  | 13e | Describe any methods used to explore possible causes of heterogeneity among study results (e.g. subgroup analysis, meta-regression). | 6,7 |
|  | 13f | Describe any sensitivity analyses conducted to assess robustness of the synthesized results. | 7 |
| Reporting bias assessment | 14 | Describe any methods used to assess risk of bias due to missing results in a synthesis (arising from reporting biases). | 7 |
| Certainty assessment | 15 | Describe any methods used to assess certainty (or confidence) in the body of evidence for an outcome. | 7 |
| **RESULTS** | | |  |
| Study selection | 16a | Describe the results of the search and selection process, from the number of records identified in the search to the number of studies included in the review, ideally using a flow diagram. | 8 |
|  | 16b | Cite studies that might appear to meet the inclusion criteria, but which were excluded, and explain why they were excluded. | 8 |
| Study characteristics | 17 | Cite each included study and present its characteristics. | 8 |
| Risk of bias in studies | 18 | Present assessments of risk of bias for each included study. | 8 |
| Results of individual studies | 19 | For all outcomes, present, for each study: (a) summary statistics for each group (where appropriate) and (b) an effect estimate and its precision (e.g. confidence/credible interval), ideally using structured tables or plots. | \| Table 2, Fig 2 & S3, Table S4 & S5 \| \| --- \| |
| Results of syntheses | 20a | For each synthesis, briefly summarise the characteristics and risk of bias among contributing studies. | 8 |
|  | 20b | Present results of all statistical syntheses conducted. If meta-analysis was done, present for each the summary estimate and its precision (e.g. confidence/credible interval) and measures of statistical heterogeneity. If comparing groups, describe the direction of the effect. | 8-10 |
|  | 20c | Present results of all investigations of possible causes of heterogeneity among study results. | 9 |
|  | 20d | Present results of all sensitivity analyses conducted to assess the robustness of the synthesized results. | 10 |
| Reporting biases | 21 | Present assessments of risk of bias due to missing results (arising from reporting biases) for each synthesis assessed. | 9 (Fig S2) |
| Certainty of evidence | 22 | Present assessments of certainty (or confidence) in the body of evidence for each outcome assessed. | 9,10 |
| **DISCUSSION** | | |  |
| Discussion | 23a | Provide a general interpretation of the results in the context of other evidence. | 11,12 |
|  | 23b | Discuss any limitations of the evidence included in the review. | 12,13 |
|  | 23c | Discuss any limitations of the review processes used. | 12,13 |
|  | 23d | Discuss implications of the results for practice, policy, and future research. | 13 |
| **OTHER INFORMATION** | | |  |
| Registration and protocol | 24a | Provide registration information for the review, including register name and registration number, or state that the review was not registered. | 5 |
|  | 24b | Indicate where the review protocol can be accessed, or state that a protocol was not prepared. | 5 |
|  | 24c | Describe and explain any amendments to information provided at registration or in the protocol. | 5 |
| Support | 25 | Describe sources of financial or non-financial support for the review, and the role of the funders or sponsors in the review. | 13 |
| Competing interests | 26 | Declare any competing interests of review authors. | 13 |
| Availability of data, code and other materials | 27 | Report which of the following are publicly available and where they can be found: template data collection forms; data extracted from included studies; data used for all analyses; analytic code; any other materials used in the review. | 14 |

**Table S4**: PRISMA checklist for abstracts

| **Section and Topic** | **Item #** | **Checklist item** | **Reported (Yes/No)** |
| --- | --- | --- | --- |
| **TITLE** | | |  |
| Title | 1 | Identify the report as a systematic review. | Yes |
| **BACKGROUND** | | |  |
| Objectives | 2 | Provide an explicit statement of the main objective(s) or question(s) the review addresses. | Yes |
| **METHODS** | | |  |
| Eligibility criteria | 3 | Specify the inclusion and exclusion criteria for the review. | Yes |
| Information sources | 4 | Specify the information sources (e.g. databases, registers) used to identify studies and the date when each was last searched. | Yes |
| Risk of bias | 5 | Specify the methods used to assess risk of bias in the included studies. | Yes |
| Synthesis of results | 6 | Specify the methods used to present and synthesise results. | Yes |
| **RESULTS** | | |  |
| Included studies | 7 | Give the total number of included studies and participants and summarise relevant characteristics of studies. | Yes |
| Synthesis of results | 8 | Present results for main outcomes, preferably indicating the number of included studies and participants for each. If meta-analysis was done, report the summary estimate and confidence/credible interval. If comparing groups, indicate the direction of the effect (i.e. which group is favoured). | Yes |
| **DISCUSSION** | | |  |
| Limitations of evidence | 9 | Provide a brief summary of the limitations of the evidence included in the review (e.g. study risk of bias, inconsistency and imprecision). | Yes |
| Interpretation | 10 | Provide a general interpretation of the results and important implications. | Yes |
| **OTHER** | | |  |
| Funding | 11 | Specify the primary source of funding for the review. | Yes |
| Registration | 12 | Provide the register name and registration number. | Yes |

**Table S5**: Characteristics of 61 included trials contributing data to the network meta-analysis

*A (low risk of bias), B (risk of bias unclear), antiHTN (antihypertensive), BP (blood pressure), C (high risk of bias), cHTN (chronic hypertension), dBP (diastolic BP), gov (government), HDP (hypertensive disorder of pregnancy), LT control (‘less tight’ BP control), NA (not applicable), None (no antihypertensive therapy), NS (not specified), PET (pre-ecampsia/eclampsia), PIH (pregnancy-induced hypertension), reg (registration), sBP (systolic BP), T control (‘tight’ BP control), T1 (first trimester), T2 (second trimester), T3 (third trimester)*

** These were trials of differential BP control.*

|  |  |  |  | **BP** | |  |  | **Control arm** | |  | **Treatment arm** | | |  |  |  |  |  |  | **BP (mmHg)** | | | **Risk of bias** | | | | | | |
| --- | --- | --- | --- | --- | --- | --- | --- | --- | --- | --- | --- | --- | --- | --- | --- | --- | --- | --- | --- | --- | --- | --- | --- | --- | --- | --- | --- | --- | --- |
| **Study (1^st^ author)** | **Publicatinon year** | **Developed country** | **N women** | sBP | dBP | **HDP** | **Korotkoff for dBP** | Placebo/no therapy | Drug | **N trial arms** | 1 | 2 | 3 | **Placebo/no therapy** | **GA at entry** | **Publication type** | **Unpublished data** | **Prospective reg** | **Funding** | Threshold  (Treatment arm) | Threshold (placebo/ no therapy arm) for additional antiHTN | Target | OVERALL | Random sequence generation | Concealment | Blinding | Blinding outcome assessment | Incomplete data | Selective reporting |
| Argentina (Voto) 1985 | 1985 | No | 60 | ≥160 | ≥100 | NS | NS | No | Yes | 2 | Atenolol | Methyldopa | - | - | T3 | Full paper | No | NS | NS | dBP ≥100 | NA | NS | B | NS | NS | Open label | No | No | Published only |
| Argentina (Voto) 1987 | 1987 | No | 20 | ≥160 | ≥100 | NS | NS | No | Yes | 2 | Ketanserin | Methyldopa | - | - | T3 | Full paper | No | NS | NS | dBP ≥100 | NA | NS | B | NS | NS | Open label | No | No | Published only |
| Argentina (Casavilla) 1988 | 1988 | No | 36 | ≥140 | ≥90 | cHTN | NS | No | Yes | 2 | Mepindolol | Methyldopa | - | - | T1 | Abstract | No | NS | NS | ≥140/90 | NA | Optimal  response | B | NS | NS | Open label | No | No | Published only |
| Australia (Livingstone) 1983 | 1983 | Yes | 28 | ≥140 | ≥90 | PIH | NS | No | Yes | 2 | Propran-olol | Methyldopa | - | - | T2-T3 | Full paper | No | NS | NS | ≥140/ 90 | NA | NS | B | NS | NS | Open label | No | No | Published only |
| Australia (Gallery) 1985a | 1985 | Yes | 183 | NS | ≥90 | NS | IV | No | Yes | 2 | Oxpren-olol | Methyldopa | - | - | T3 | Full paper | No | NS | NS | dBP ≥90 | NA | dBP <80 | B | Series random numbers | NS | Open label | No | No | Published only |
| Australia (Davis) 2001 | 2001 | Yes | 16 | NS | NS | PIH | NS | Yes | No | 2 | GTN patches | - | - | Placebo | T2-T3 | Letter | Yes | NS | NS | NS | NS | NS | B | Central telephone | Telephone | Single blind | No | No | Published only |
| Brazil (Kahhale) 1985 | 1985 | No | 100 | ≥140 | ≥90 | cHTN | NS | Yes | No | 2 | Pindolol | - | - | None | T2 | Full paper | No | NS | NS | ≥140/90 | NS | BP stabili-  sation | B | NS | NS | Open label | No | No | Published only |
| Brazil (Freire) 1988 | 1988 | No | 40 | NS | ≥95 | cHTN | NS | No | Yes | 2 | Pindolol | Methyldopa | - | - | T3 | Full paper | Yes | NS | NS | dBP ≥85 | NA | Lack of satisfactory control | B | NS | Consecutive boxes | Open label | No | No | Published and unpublished |
| Brazil (Nascime) 2000a | 2000 | No | 199 | NS | ≥90 | cHTN | V | Yes | No | 2 | Verapamil | - | - | Placebo | T2 | Full thesis | Yes | NS | NS | ≥140/90 | NS | “BP control” | A | NS | Consecutive boxes | Double blind | Yes | 7.5% | Published only |
| Brazil (Trapini) 2016 | 2016 | No | 100 | ≥140 | ≥90 | PET | IV | Yes | No | 2 | Sildenafil | - | - | Placebo | T2-T3 | Full paper | No | NS | Non commercial. | ≥140/90 | NS | NS | A | NS (but blocked) | Sealed envelopes | Double blind | Yes | 7.0% | Published only |
| Caribbean Is (Plouin) 1990 | 1990 | No | 155 | NS | ≥85 | PIH | IV | Yes | No | 2 | Oxprenolol + Dihydralazine | - | - | Placebo | T3 | Full paper | Yes | NS | Industry. | dBP ≥85 | dBP >105 | dBP <86 | A | NS (but blocked) | Consecutive boxes | Double blind | Yes | 0.6% | Published and unpublished |
| France (Plouin) 1987 | 1987 | Yes | 188 | NS | ≥90 | cHTN | IV | No | Yes | 2 | Labetalol | Methyldopa | - | - | T2-T3 | Full paper | No | NS | Industry. | dBP ≥90 | NA | DBP <86mmHg | B | NS (but blocked) | Sealed envelopes | Open label | No | 6.4% | Published only |
| France (Lardoux) 1988a | 1988 | Yes | 63 | NS | ≥90 | NS | NS | No | Yes | 3 | Labetalol | Methyldopa | Acebutolol | - | T2 | Full paper | No | NS | NS | dBP ≥90 | NA | NS | B | NS | NS | Open label | No | No | Published only |
| France (Jannet) 1994 | 1994 | Yes | 100 | ≥140 | ≥90 | NS | NS | No | Yes | 2 | Nicardipine | Metoprolol | - | - | T2-T3 | Full paper | No | NS | NS | ≥140/90 | NA | NS | B | Computer generated | Sealed envelopes | Open label | No | No | Published only |
| Hong Kong (Li) 1990 | 1990 | Yes | 41 | ≥140 | ≥90 | PET | NS | Yes | No | 2 | Labetalol | - | - | Placebo | NS | Abstract | No | NS | NS | ≥140/90 | BP>165/105 | NS | B | NS | NS | Double blind | Yes | No | Published only |
| India (Oumachigui) 1992 | 1992 | No | 30 | ≥140 | ≥90 | PIH | NS | No | Yes | 2 | Metoprolol | Methyldopa | - | - | T3 | Full paper | No | NS | Industry. | ≥140/90 | NA | dBP <86 | B | NS | NS | Open label | No | No | Published only |
| India (Banerjee) 2002 | 2002 | No | 111 | ≥140 | ≥90 | PIH/ PET | NS | No | Yes | 2 | Nimodipine | Methyldopa | - | - | T2-T3 | Full paper | No | NS | NS | ≥140/90 | NA | NS | B | NS | NS | Open label | No | 6% | Published only |
| India (Molvi) 2012 | 2012 | No | 150 | ≥140 | ≥90 | PIH | V | Yes | Yes | 3 | Labetalol | Methyldopa | = | None | T2-T3 | Full paper | No | NS | University | ≥140/90 | dBP >105 | >110/ 70 &  <140/ 90 | **C** | Manually shuffled | Sealed envelopes | Open label | No | 0.6% | Published only |
| India (Aparna) 2013b | 2013 | No | 100 | ≥150 | ≥100 | PIH | V | No | Yes | 2 | Nifedipine | Methyldopa | - | - | T3 | Full paper | No | NS | NS | ≥150/ 100 | NA | dBP 80-100 | B | Random numbers | NS | Open label | No | 8% | Published only |
| India (Babbar) 2015a | 2015 | No | 240 | ≥140 | ≥90 | PIH | IV | No | Yes | 3 | Labetalol | Methyldopa | Nifedipine | - | T2-T3 | Full paper | No | NS | NS | ≥140/90 | NA | “BP control” | B | NS | NS | Open label | No | No | Published only |
| Ireland (Blake) 1991 | 1991 | Yes | 36 | ≥140 | ≥90 | NS | V | Yes | No | 2 | Atenolol and/or Methyldopa |  |  | None | NS | Full paper | Yes | NS | NS | ≥140/90 | 140/90 | “Average normal” BP | **C** | Manually shuffled | Sealed envelopes | Open label | No | No | Published only |
| Israel (Ellenboggen) 1986a | 1986 | Yes | 32 | NS | ≥90 | PIH | NS | No | Yes | 2 | Pindolol | Methyldopa |  | - | T3 | Full paper | No | NS | NS | dBP ≥90 | NA | NS | B | NS | NS | Open label | No | No | Published only |
| Israel (Rosenfeld) 1986b | 1986 | Yes | 44 | ≥150 | ≥90 | NS | NS | No | Yes | 2 | Pindolol + Hydralazine | Hydralazine | - | - | T2-T3 | Full paper | No | NS | NS | ≥150/90 | NA | BdP ≤90 | B | NS | NS | Open label | No | No | Published only |
| Israel (Bott-Kanner) 1992a | 1992 | Yes | 60 | NS | ≥85 | NS | IV | Yes | No | 2 | Pindolol | - | - | Placebo | T2-T3 | Full paper | No | NS | NS | DBP 85-99 | dBP 100-109 | dBP < 85 | A | Random numbers | Consecutive boxes | Double blind | Yes | No | Published only |
| Israel (Paran) 1995 | 1995 | Yes | 51 | ≥140 | ≥95 | NS | V | No | Yes | 3 | Propranolol + Hydralazine | Pindolol + hydralazine | Hydralazine | - | T3 | Full paper | No | NS | NS | 140-160/95-110 | NS | ≤140/90 | B | NS | NS | Open label | No | No | Published only |
| Italy (Catalano) 1997 | 1997 | Yes | 100 | ≥140 | ≥90 | PET | NS | Yes | No | 2 | Nifedipine |  |  | None | T3 | Full paper | No | NS | NS | ≥140/90 | NS | ≤140/90 | B | NS | NS | Open label | No | No | Published only |
| Italy (Fruppo di Studio) 1998 | 1998 | Yes | 283 | NS | ≥90 | PIH/ PET | NS | Yes | No | 2 | Nifedipine |  |  | None | T2-T3 | Full paper | No | YES | University | dBP ≥90 | dBP >110 | NS | B | Computer generated | Telephone | Open label | No | 7.80% | Published only |
| Italy (Neri) 1999 | 1999 | Yes | 36 | ≥140 | ≥90 | PIH/ PET | IV | No | Yes | 3 | GTN | Nifedipine | - | - | T3 | Full paper | Yes | NS | NS | ≥140/90 | NA | NS | B | NS | sealed envelopes | Open label | No | 16.6% | Published & unpublished |
| Italy (Borghi) 2000 | 2000 | Yes | 20 | NS | NS | PET | NS | No | Yes | 2 | Nifedipine | Methyldopa | - | - | NS | Full paper | Yes | NS | NS | NS | NA | NS | B | NS | Consecutive boxes | Single blind | No | No | Published & unpublished |
| Pakistan (Sharif) 2016 | 2016 | No | 314 | ≥140 | ≥100 | PIH | NS | No | Yes | 2 | Labetalol | Methyldopa | - | - | T3 | Full paper | No | NS | NS | ≥140/90 | NA | dBP <90 | B | Lottery method | NS | Open label | No | 1.3% | Published only |
| Panama (Vigil-De Gracia) 2014 | 2014 | No | 63 | ≥140 | ≥90 | cHTN | NS | No | Yes | 3 | Amlodipine | Furosemide | - | ASA 75mg/d | T2 | Full paper | No | NS | NS | ≥140/90 | NA | NS | B | Computer generated | Sealed envelopes | Open label | No | 5.0% | Published only |
| South Africa (Odendaal), 1991a | 1991 | No | 32 | ≥140 | ≥90 | PIH | NS | Yes | No | 2 | Prazosin | - | - | Placebo | T2-T3 | Full report | No | NS | NS | ≥140/90 | >160/110 | 130/80 to  140/90 | B | NS | Sealed envelopes | NS | NS | No | Published only |
| South Africa (Eloff) 1993 | 1993 | No | 29 | NS | ≥90 | NS | NS | No | Yes | 2 | Nifedipine | Methyldopa | - | - | T3 | Abstract | No | NS | NS | dBP ≥90 | NA | “To control BP” | B | NS | NS | Open label | No | 10.3% | Published only |
| Sudan (Hassan) 2002 | 2002 | No | 70 | NS | ≥90 | PET | IV | Yes | No | 2 | Methyldopa | - | - | None | T3 | Full paper | No | NS | NS | dBP ≥90 | dBP >110 | dBP <90 | B | NS | NS | Open label | No | No | Published only |
| Sweden (Wichman) 1984 | 1984 | Yes | 52 | ≥140 | ≥90 | NS | V | Yes | No | 2 | Metoprolol | - | - | Placebo | T3 | Full paper | Yes | NS | Charity | ≥140/90 | 140/90 | <140/90 | A | NS | Telephone | Double blind | Yes | No | Published & unpublished |
| Sweden (Högstedt) 1985 | 1985 | Yes | 168 | NS | ≥90 | PIH | V | Yes | No | 2 | Metoprolol + Hydralazine | - | - | None | T2-T3 | Full paper | Yes | NS | NS | dBP ≥90 | dBP >110 | dBP < 90 | B | NS | Envelopes (no details) | Open label | No | 4.0% | Published only |
| Sweden (Swensson) 1995 | 1995 | Yes | 118 | NS | ≥95 | NS | IV | Yes | No | 2 | Isradipine | - | - | Placebo | T3 | Full paper | No | NS | NS | dBP 95 -110 | dBP >110 | NS | B | NS | NS | Double blind | Yes | 6.0% | Published only |
| UK (Leather) 1968 | 1968 | Yes | 100 | NS | ≥90 | NS | NS | Yes | No | 2 | Methyldopa + benfluorazide | - | - | None | NS | Full paper | No | NS | Non commercial | dBP ≥90 | NS | NS | B | NS | NS | Open label | No | No | Published only |
| UK (Redman) 1976 | 1976 | Yes | 247 | ≥140 | ≥90 | NS | IV | Yes | No | 2 | Methyldopa | - | - | None | T2-T3 | Full paper | No | NS | Industry. | ≥140/90 to 28w,  ≥ 150/95 after 28w | >170/110 | “To achieve good BP control” | B | NS | NS | Open label | No | 2.00% | Published only |
| UK (Lamming) 1980 | 1980 | Yes | 26 | NS | NS | PIH | IV | No | Yes | 2 | Labetalol | Methyldopa | - | - | T3 | Full paper | No | NS | Industry. | **not reported** | NA | MAP < 103.3 | B | Random numbers | NS | Open label | No | No | Published only |
| UK (Walker) 1982 | 1982 | Yes | 126 | NS | ≥95 | PIH/ PET/ cHTN | NS | Yes | No | 2 | Labetalol | - | - | None | T2-T3 | Full report | Yes | NS | Industry. | dBP ≥95 | NS | “BP control” | B | NS | Envelopes (no details) | Open label | No | No | Published & unpublished |
| UK (Rubin) 1983a | 1983 | Yes | 120 | ≥140 | >/=90 | PIH/ PET | IV | Yes | No | 2 | Atenolol | - | - | Placebo | T3 | Full paper | No | NHS | Industry. | ≥140/90 | >170/110 | dBP <90 | B | NS | NS | Double blind | Yes | No | Published only |
| UK (Fidler) 1983b | 1983 | Yes | 100 | NS | ≥95 | PIH/ PET/ cHTN | IV | No | Yes | 2 | Oxprenolol | Methyldopa | - | - | T3 | Full paper | No | NS | Industry. | dBP ≥95 | NA | dBP < 95 | B | NS | NS | Open label | No | 4.00% | Published only |
| UK (Thorley) 1984 | 1984 | Yes | 60 | NS | NS | NS | V | No | Yes | 2 | Atenolol | Methyldopa | - | - | T2-T3 | Full paper | No | NS | NS | NS | NS | NS | B | NS | NS | Open label | No | 4.0% | Published only |
| UK (Pickles) 1989 | 1989 | Yes | 152 | ≥140 | ≥90 | PIH | IV | Yes | No | 2 | Labetalol | - | - | Placebo | T2-T3 | Full paper | No | NS | Industry. | ≥140/90 | Inadequate  BP control | “BP control” | A | Random numbers | Consecutive boxes | Double blind | Yes | 5.3% | Published only |
| UK (Butter) 1990 | 1990 | Yes | 33 | ≥140 | ≥90 | cHTN | V | Yes | No | 2 | Atenolol | - | - | Placebo | T2 | Full paper | Yes | NHS | Industry. | ≥140/90 | NS | <140/90 or  200mg atenolol | B | NS | NS | Double blind | Yes | 12.1% | Published & unpublished |
| UK (Cruickshank) 1992 | 1992 | Yes | 114 | NS | ≥90 | PIH | IV | Yes | No | 2 | Labetalol | - | - | None | T3 | Full paper | Yes | NS | Industry. | dBP ≥90 | NS | DBP < 90mmHg | B | NS | Sealed envelopes | Open label | No | No | Published & unpublished |
| UK (Samangaya) 2009 | 2009 | Yes | 30 | NS | ≥90 | PIH/ PET/ cHTN | NS | Yes | No | 2 | Sildenafil | - | - | Placebo | T2-T3 | Full paper | No | Yes | Industry. | dBP ≥90 | At clinician discretion | NS | B | Computer generated | Telephone | Double blind | Yes | 10.3% | Published only |
| UK (Webster) 2017 | 2017 | Yes | 114 | ≥140 | ≥90 | cHTN | NS | No | Yes | 2 | Labetalol | Nifedipine | - | None | T2-T3 | Full paper | No | Yes | Nonncommercial | ≥140/90 | NA | dBP < 85 | B | Computer generated | Computer system | Open label | NS | 1.8% | Published only |
| USA (Arias) 1979 | 1979 | Yes | 58 | ≥140 | ≥90 | cHTN | NS | Yes | No | 2 | Methyldopa and/or hydral and/or HCTZ | - | - | None | T2 | Full paper | No | NS | NS | ≥140/90 | NS | dBP <90 |  | NS | NS | Open label | No | No | Published only |
| USA (Sibai) 1987a | 1987 | Yes | 200 | ≥140 | ≥90 | PET | NS | Yes | No | 2 | Labetalol | - | - | None | T3 | Full paper | No | NS | NS | ≥140/90 | Clinician discretion | dBP <90 | B | Computer generated | Sealed envelopes | Open label | No | 0.1% | Published only |
| USA (Weitz) 1987b | 1987 | Yes | 25 | ≥140 | ≥90 | cHTN | NS | Yes | No | 2 | Methyldopa | - | - | Placebo | NS | Full paper | No | NS | Industry. | ≥140/90 | NS | BP ≤140/90 | B | NS | NS | Double blind | Yes | No | Published only |
| USA (Sibai) 1990a | 1990 | Yes | 300 | NS | NS | cHTN | NS | Yes | Yes | 3 | Labetalol | Methyldopa | - | None | T1 | Full paper | Yes | NS | NS | NS | >160/110 | NS | B | Computer generted | Sealed envelopes | Open label | No | 12.0% | published and unpublished |
| USA (Sibai) 1992 | 1992 | Yes | 200 | ≥140 | ≥9 | pet | NS | Yes | No | 2 | Nifedipine | - | - | None | T3 | Full paper | No | NS | NS | ≥140/90 | Clinician discretion | <140/90 | B | Computer generated | Sealed envelopes | Open label | No | 1.5% | Published only |
| Venezuela (Faneite) 1988 | 1988 | No | 31 | ≥140 | ≥90 | PIH/ cHTN | NS | No | Yes | 2 | Mepindolol | Methyldopa | - | - | T2-T3 | Full paper | No | NS | Industry. | ≥140/90 | NA | <140/90 | B | Random numbers | NS | Open label | No | No | Published only |
| Australia (Horvath) 1985b | 1985 | Yes | 100 | >130 | >85 | PIH/ cHTN | NS | No | Yes | 2 | Clonidine hydrochloride | Methyldopa | - | - | T2-T3 | Full paper | No | NS | Industry & gov | >135/85 | NA | NS | B | NS | NS | Double blind | Yes | 5.0% | Published only |
| Finland (Tuimala) 1988b | 1988 | Yes | 51 | ≥150 | ≥95 | NS | NS | No | Yes | 2 | Pindolol | Atenolol |  | - | T2-T3 | Full paper | No | NS | NS | ≥150/95 | NA | NS | B | Random number table | NS | Open label | NS | no | Published only |
| Sweden (Montan) 1992 | 1992 | Yes | 29 | ≥140 | ≥90 | PIH/ PET/ cHTN | V | No | Yes | 2 | Atenolol | Pindolol | - | - | T3 | Full paper | No | NS | Non commercial | ≥140/90 | NA | NS | B | NS | Sealed envelopes | Double blind | Yes | 9.4% | Published only |
| Egypt (El-Guindy) 2008* | 2008 | No | 120 | 140-159 | 90-99 | PIH/ cHTN | V | No | Yes | 2 | Methyldopa  (very tight <130/80) | Methyldopa  (tight 130-139/80-89) | - | - | T3 | Full paper | No | NS | NS | ≥140/90 | NA | <130/80 vs.  130-139/80-89 | A | Computer generated | Sealed opaque envelopes | Double blind | Yes | 4.0% | Published only |
| Canada (Magee) 2007* | 2007 | Yes | 132 | <170 | 90-109 | PIH/chronic | V | Yes | No | 2 | T control  (most often labetalol) | - | LT control | - | T2-T3 | Full paper | No | NS | Non commercial | dBP 90-109 | dBP >105 but could consider at 101-105 | T: dBP 85  LT: dBP 100 | A | Computer generated | Telephone | Open label | Yes | 0.8% | Published only |
| Canada (Magee) 2015* | 2015 | Yes | 987 | <160 | dBP 85-105 if on meds, 90-105 if not | PIH/chronic | V | Yes | No | 2 | T control  (most often labetalol) | - | - | LT control | T2-T3 | Full paper | No | Yes | Non commercial | dBP 90-105 | dBP ≥105 or sBP ≥160 regardless of dBP | LT: dBP 100  T: dBP 85 | A | Computer generated | Telephone | Open label | Yes | 4.2% | Published only |

**Table S6**: Characteristics of included trials by main outcomes for primary analysis

*SGA (small-for-gestational age)*

** Treatments considered were placebo/no therapy, and the antihypertensives labetalol, methyldopa, calcium channel blockers, beta-blockers, and multi-drug.*

*ł Two three-arm trials informed both drug vs. placebo/no therapy and drug vs. drug comparisons.*

|  | **Severe hypertension** | **Proteinuria/**  **preeclampsia** | **Perinatal death** | **SGA** | **Preterm birth** | **Admission to**  **neonatal care unit** |
| --- | --- | --- | --- | --- | --- | --- |
| N studies reporting outcome | 32 | 32 | 44 | 26 | 27 | 16 |
| Two-arm trials | 30 | 29 | 40 | 24 | 25 | 15 |
| Three-arm trials | 2 | 3 | 4 | 2 | 2 | 1 |
| N participants | 3811 | 4662 | 5051 | 3848 | 4283 | 2909 |
| N treatments considered* | 6 | 6 | 6 | 6 | 6 | 6 |
| N possible pairwise comparisons | 15 | 15 | 15 | 15 | 15 | 15 |
| N with direct data | 13 | 15 | 9 | 13 | 12 | 12 |
| N drug vs. placebo/no therapy | 9ł | 7 | 5 | 7 | 6 | 6 |
| N drug vs drug | 6ł | 8 | 10 | 6 | 6 | 6 |
| N events | 599 (15.7%) | 688 (14.8%) | 226 (4.5%) | 482 (12.5%) | 842 (19.6%) | 557 (19.1%) |
| Median event rate (IQR) | 13%  (7%, 22%) | 14%  (8%, 21%) | 3%  (0%, 6%) | 12%  (7%, 17%) | 16%  (11%, 26%) | 18%  (12%, 24%) |
| N studies by N events in each arm |  |  |  |  |  |  |
| ≥1 event in each arm | 27/32 | 30/32 | 23/44 | 21/26 | 25/27 | 15/16 |
| ≥1 arm with no events | 5/30 | 2/34 | 21/23 | 5/26 | 2/24 | 1/16 |
| No event in any arm | 0 | 0 | 12/23 | 1/26 | 0 | 0 |
| Median N events (IQR) | 6 (2,11) | 7 (4,14) | 1 (0,3) | 6 (4,12) | 9 (5,17) | 13 (7,20) |

**Table S7**: Sensitivity analyses of main outcomes for antihypertensives vs. placebo/no therapy, in comparison with results from the primary analysis (OR and 95% CI)

*CI (credible interval), OR (odds ratio)*

** Sensitivity analysis 1 includes all data in the primary analysis, plus three additional trials of differential BP control.*

*** Sensitivity analysis 2 includes all data in the primary analysis, plus all other trials of additional drugs that were not of primary interest in the network. Only results for drugs of interest are presented, as additional estimates were not informative.*

**** Sensitivity analysis 3 excludes two trials at high risk of bias (Molvi et al 2012 [PMID: 22249781] and Blake et al 1991 [PMID: 2021561])*

| **Drug** | **Severe hypertension** | | | | **Proteinuria** | | | | **Perinatal death** | | | | **Small for gestational age** | | | | **Preterm birth** | | | | **Admission to NICU** | | | |
| --- | --- | --- | --- | --- | --- | --- | --- | --- | --- | --- | --- | --- | --- | --- | --- | --- | --- | --- | --- | --- | --- | --- | --- | --- |
|  | Pri-  mary | Sensitivity 1* | Sensitivity 2** | Sensitivity 3*** | Pri- mary | Sensitivity 1* | Sensitivity 2** | Sensitivity 3*** | Pri-mary | Sensitivity 1* | Sensitivity 2** | Sensitivity 3*** | Pri-mary | Sensitivity 1* | Sensitivity 2** | Sensitivity 3*** | Primary | Sensitivity 1* | Sensitivity 2** | Sensitivity 3*** | Primary | Sensitivity 1* | Sensitivity 2** | Sensitivity 3*** |
| Placebo | 1.00 (ref) | 1.00 (ref) | 1.00 (ref) | 1.00 (ref) | 1.00 (ref) | 1.00 (ref) | 1.00 (ref) | 1.00 (ref) | 1.00 (ref) | 1.00 (ref) | 1.00 (ref) | 1.00 (ref) | 1.00 (ref) | 1.00 (ref) | 1.00 (ref) | 1.00 (ref) | 1.00 (ref) | 1.00 (ref) | 1.00 (ref) |  | 1.00 (ref) | 1.00 (ref) | 1.00 (ref) |  |
| Labetalol | 0.31 (0.17, 0.52) | 0.31 (0.17, 0.53) | 0.31 (0.17, 0.52) | 0.35 (0.19, 0.65) | 0.73 (0.54, 0.99) | 0.74 (0.55, 1.00) | 0.73 (0.55, 1.02) | 0.87 (0.64,1.20) | 0.54 (0.27, 1.01) | 0.55 (0.28, 1.05) | 0.56 (0.28, 1.03) | 0.51 (0.24, 1.29) | 1.01 (0.72, 1.46) | 1.00 (0.70, 1.44) | 1.00 (0.70, 1.45) | 1.20 (0.81,1.80) | 0.85 (0.62, 1.15) | 0.86 (0.63, 1.16) | 0.84 (0.63, 1.14) | 1.01 (0.74,1.41) | 0.96 (0.64, 1.41) | 0.97 (0.65, 1.42) | 0.96 (0.65, 1.38) | 1.19 (0.79,1.84) |
| Methyldopa | 0.60 (0.40, 0.91) | 0.60 (0.40, 0.92) | 0.60 (0.40, 0.91) | 0.59 (0.39, 1.02) | 1.12 (0.77, 1.62) | 1.13 (0.80, 1.57) | 1.11 (0.78, 1.57) | 1.24 (0.86,1.83) | 0.65 (0.34, 1.17) | 0.65 (0.35, 1.16) | 0.65 (0.37, 1.17) | 0.66 (0.36, 1.38) | 0.99 (0.65, 1.55) | 1.01 (0.64, 1.55) | 1.01 (0.67, 1.53) | 1.21 (0.67,2.15) | 0.99 (0.71, 1.42) | 1.01 (0.73, 1.43) | 1.00 (0.70, 1.42) | 1.28 (0.84,1.95) | 1.24 (0.84, 2.13) | 1.26 (0.82, 1.96) | 1.23 (0.82, 1.89) | 1.53 (0.92,2.70) |
| BB | 0.53 (0.34, 0.79) | 0.52 (0.34, 0.81) | 0.52 (0.34, 0.79) | 0.51 (0.31, 0.83) | 0.88 (0.54, 1.43) | 0.89 (0.55, 1.42) | 0.88 (0.56, 1.35) | 0.92 (0.59,1.51) | 0.76 (0.35, 1.82) | 0.74 (0.30, 1.76) | 0.78 (0.34, 1.62) | 0.77 (0.33, 1.89) | 1.27 (0.63, 2.60) | 1.25 (0.64, 2.58) | 1.25 (0.66, 2.53) | 1.33 (0.67,2.66) | 0.88 (0.51, 1.59) | 0.90 (0.51, 1.56) | 0.94 (0.52, 1.63) | 1.02 (0.55,2.08) | 1.04 (0.45, 2.48) | 1.05 (0.45, 2.54) | 1.01 (0.44, 2.40) | 1.23 (0.54,3.01) |
| CCB | 0.59 (0.37, 0.89) | 0.59 (0.36, 0.90) | 0.59 (0.37, 0.87) | 0.57 (0.33, 0.89) | 1.18 (0.84, 1.64) | 1.17 (0.85, 1.64) | 1.14 (0.85, 1.57) | 1.22 (0.87,1.67) | 0.73 (0.35, 1.40) | 0.71 (0.40, 1.56) | 0.71 (0.35, 1.43) | 0.71 (0.34, 1.78) | 0.85 (0.56, 1.29) | 0.84 (0.55, 1.31) | 0.86 (0.56, 1.29) | 0.88 (0.58,1.36) | 1.17 (0.63, 2.16) | 1.05 (0.78, 1.38) | 1.03 (0.80, 1.36) | 1.07 (0.82,1.42) | 1.04 (0.63, 1.60) | 1.04 (0.65, 1.60) | 1.04 (0.65, 1.59) | 1.13 (0.70,1.75) |
| Multi drug | 0.31 (0.11, 0.79) | 0.60 (0.34, 0.88) | 0.60 (0.36, 0.87) | 0.31 (0.10, 0.82) | 0.83 (0.47, 1.45) | 0.91 (0.68, 1.21) | 0.92 (0.69, 1.22) | 0.97 (0.56,1.66) | 1.01 (0.41, 2.40) | 0.96 (0.51, 1.76) | 0.95 (0.49, 1.75) | 0.88 (0.35, 2.38) | 0.93 (0.41, 2.12) | 1.35 (0.81, 2.03) | 1.37 (0.84, 1.99) | 0.91 (0.41,2.12) | 0.94 (0.53, 1.70) | 0.92 (0.71, 1.24) | 0.93 (0.71, 1.22) | 0.95 (0.55,1.67) | 0.65 (0.26, 1.59) | 0.98 (0.64, 1.51) | 0.99 (0.65, 1.49) | 0.66 (0.266,1.57) |

**Table S8:** Trial sequential analyses sample sizes per arm for detection of a relative risk reduction of 20%*

*CCB (calcium channel blocker)*

** These calculations assume the median event rates observed across trials, 90% power, and a superiority hypothesis, and are based on at least one direct comparison of one antihypertensive vs. another. For similar calculations for the outcome of severe hypertension, see Figure 3.*

*Ł Median event rates were taken from Table S3.*

| **Drug comparison** | **Severe hypertension** | **Proteinuria/ preeclampsia** | **Perinatal death** | **Small-for-gestational age** | **Preterm birth** | **Admission to neonatal care unit** |
| --- | --- | --- | --- | --- | --- | --- |
| Median event rate in trialsł | 13% (7%, 22%) | 14% (8%, 21%) | 3% (0%, 6%) | 12% (7%, 17%) | 16% (11%, 26%) | 18% (12%, 24%) |
| Labetalol vs. methyldopa | 5120 | 6893 | 2952 | 3420 | 2530 | 2202 |
| Labetalol vs. other beta-blocker | (No trial) | 2952 | 15,336 | 3515 | 2530 | 2202 |
| Labetalol vs. CCB | (No trial) | 2952 | 15,336 | 3515 | 2530 | 2202 |
| Beta blocker vs. CCB | 9780 | 2952 | 15,336 | (No trial) | (No trial) | 2202 |
| Beta blocker vs. methyldopa | 9780 | 2952 | 15,336 | (No trial) | 2595 | (No trial) |
| Methyldopa vs CCB | 3212 | (No trial) | 15,336 | 3515 | 2530 | 6665 |

**Figure S1**: Preferred Reporting Items for Systematic Reviews and Meta-Analyses (PRISMA) flow diagram*

** For references of key publications excluded and all those included, see Supplementary References 1 and 2, respectively.*

**Figure S2:** Network plots for main outcomes, primary analysis)

Outcomes are (from top left to bottom right): severe hypertension (Figure S2a), proteinuria (Figure S2b), perinatal death (Figure S2c), small-for-gestational age infants (Figure S2d), preterm birth (Figure S2e), and neonatal care unit admission (Figure S2f).

**
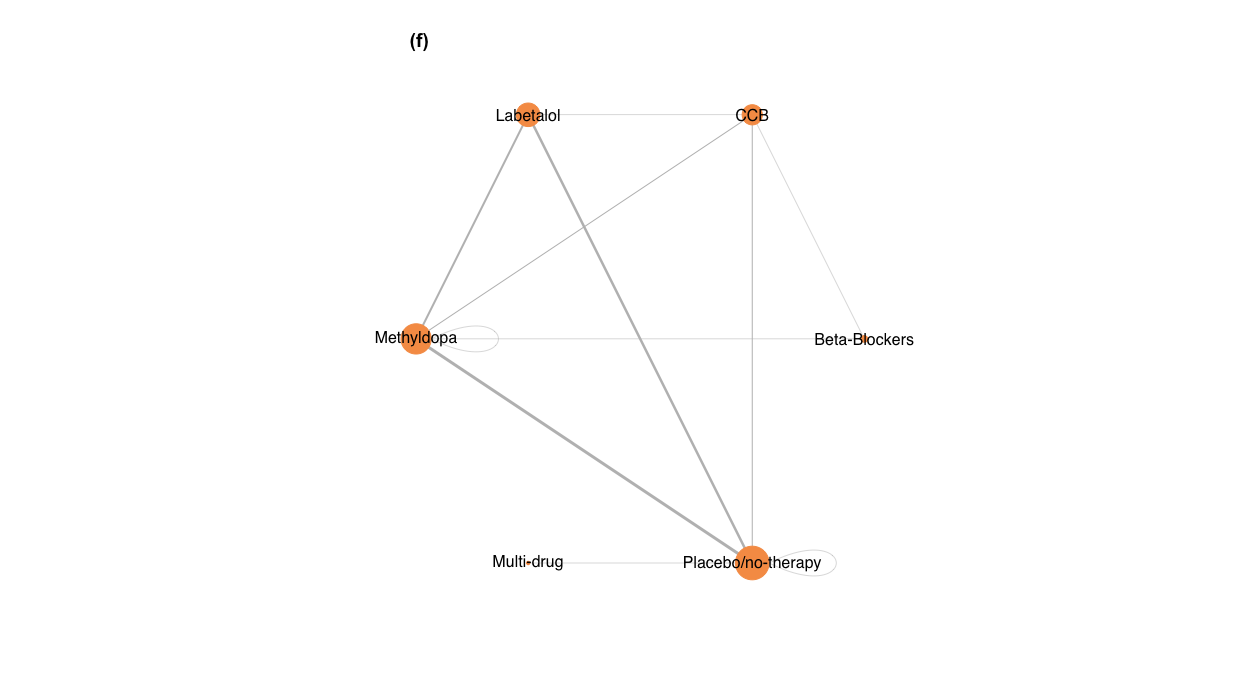

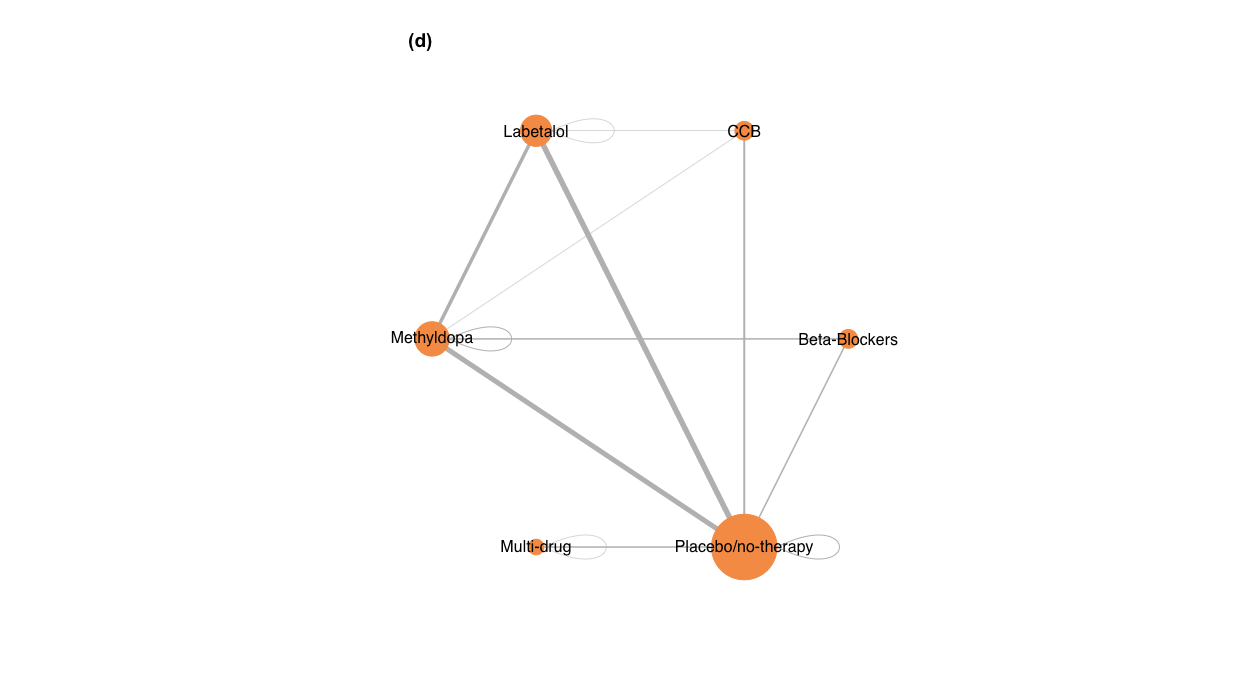

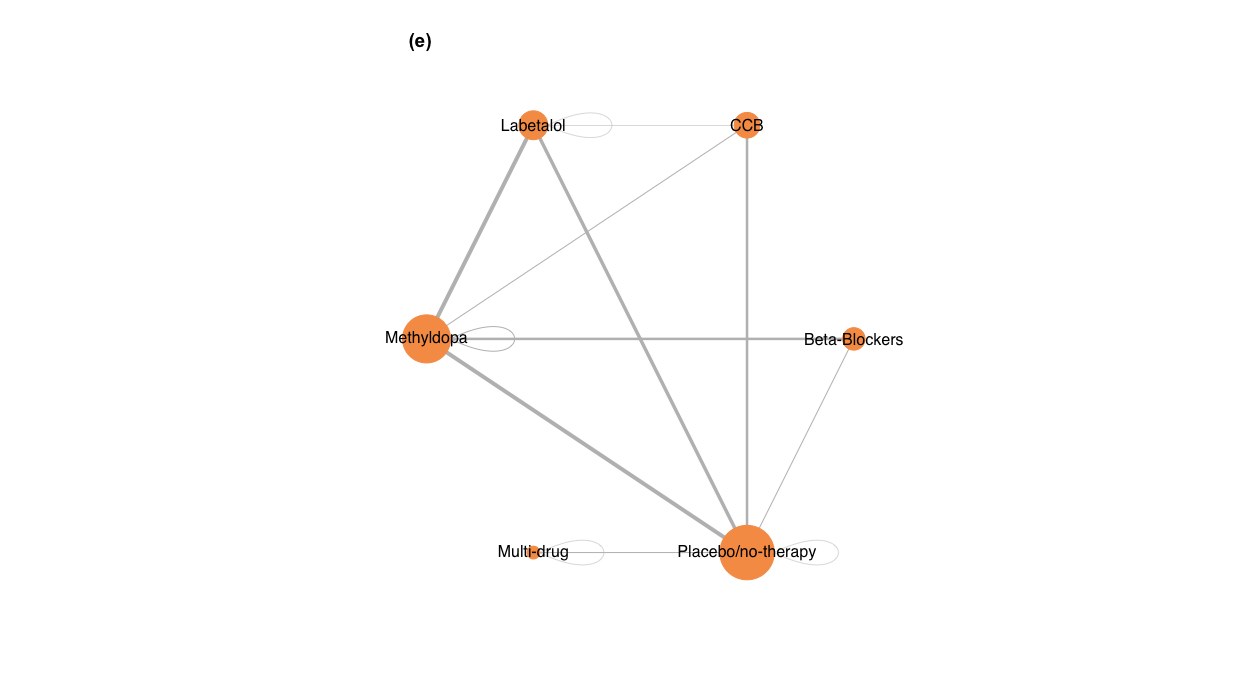

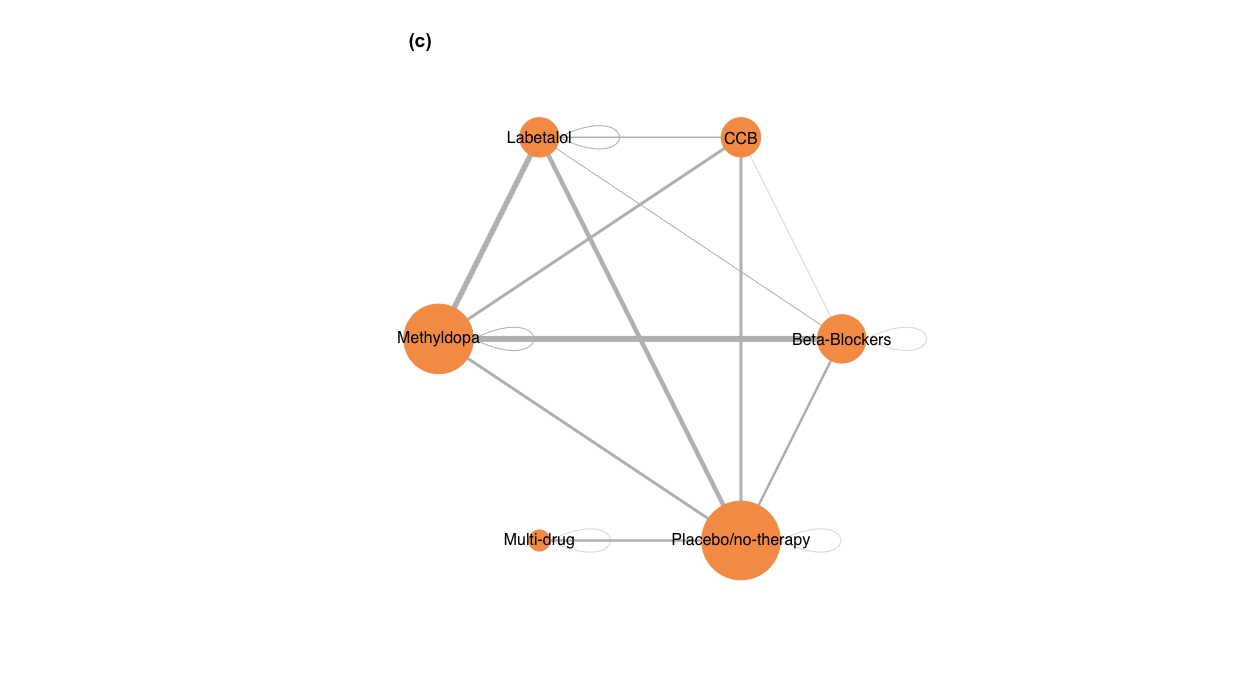
**
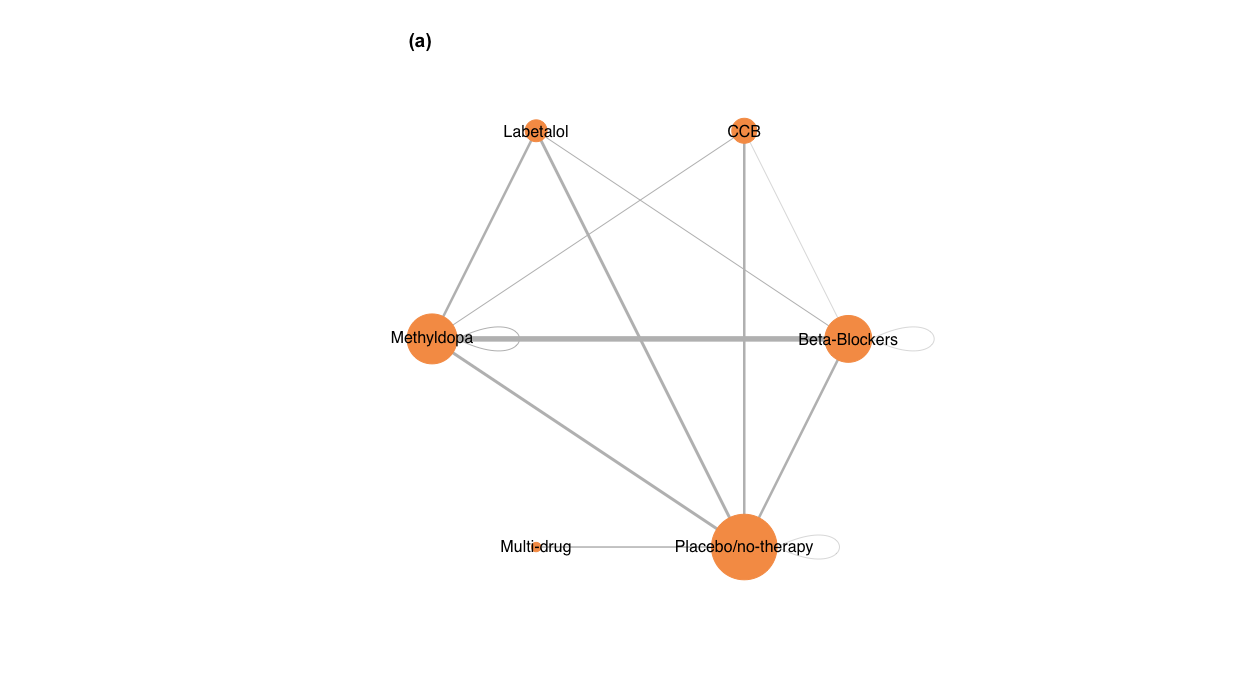
**
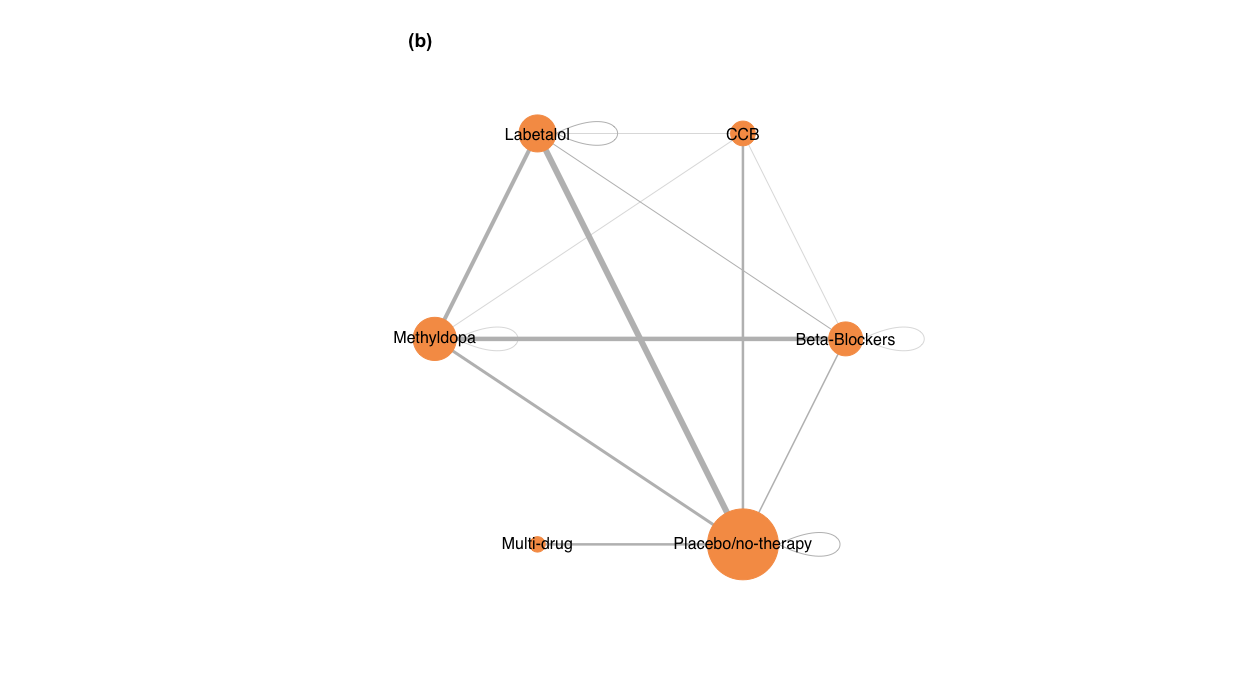
**


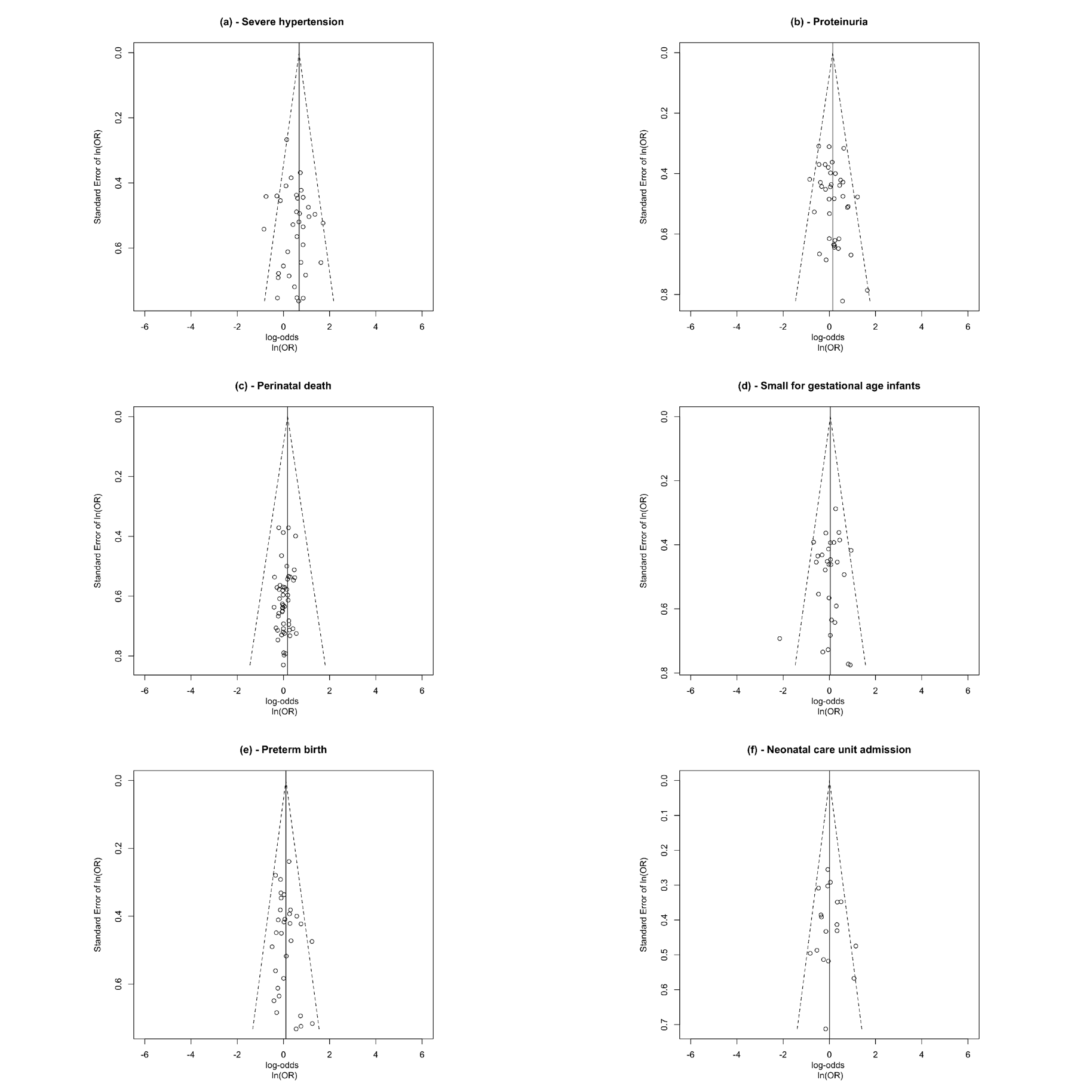
**Figure S3**: Funnel plots for the main outcomes.

All estimates of effect are odds ratios on a log scale. Outcomes are severe hypertension (A), proteinuria/preeclampsia (B), perinatal death (C), small for gestational age (D), preterm birth (E), and neonatal intensive care unit admission (F)

**Figure S4**: League tables comparing primary drugs of interest vs. placebo/no therapy and each other, for the secondary outcomes of interest.

*All estimates are odds ratios and 95% credible intervals. Outcomes are: need for additional antihypertensive therapy (A), changed/stopped drugs due to maternal side effects (B), maternal antenatal admission for more than 7 days (C), Cesarean birth (D), placental abruption (E), and respiratory distress syndrome (RDS, F). All grey squares represent comparisons that could not be reliably computed from the network due a lack of direct comparisons and sparse (or no) indirect pathways between comparators.*

*CCB (calcium channel blocker)*

**Figure S4A**: Need for additional antihypertensive therapy**
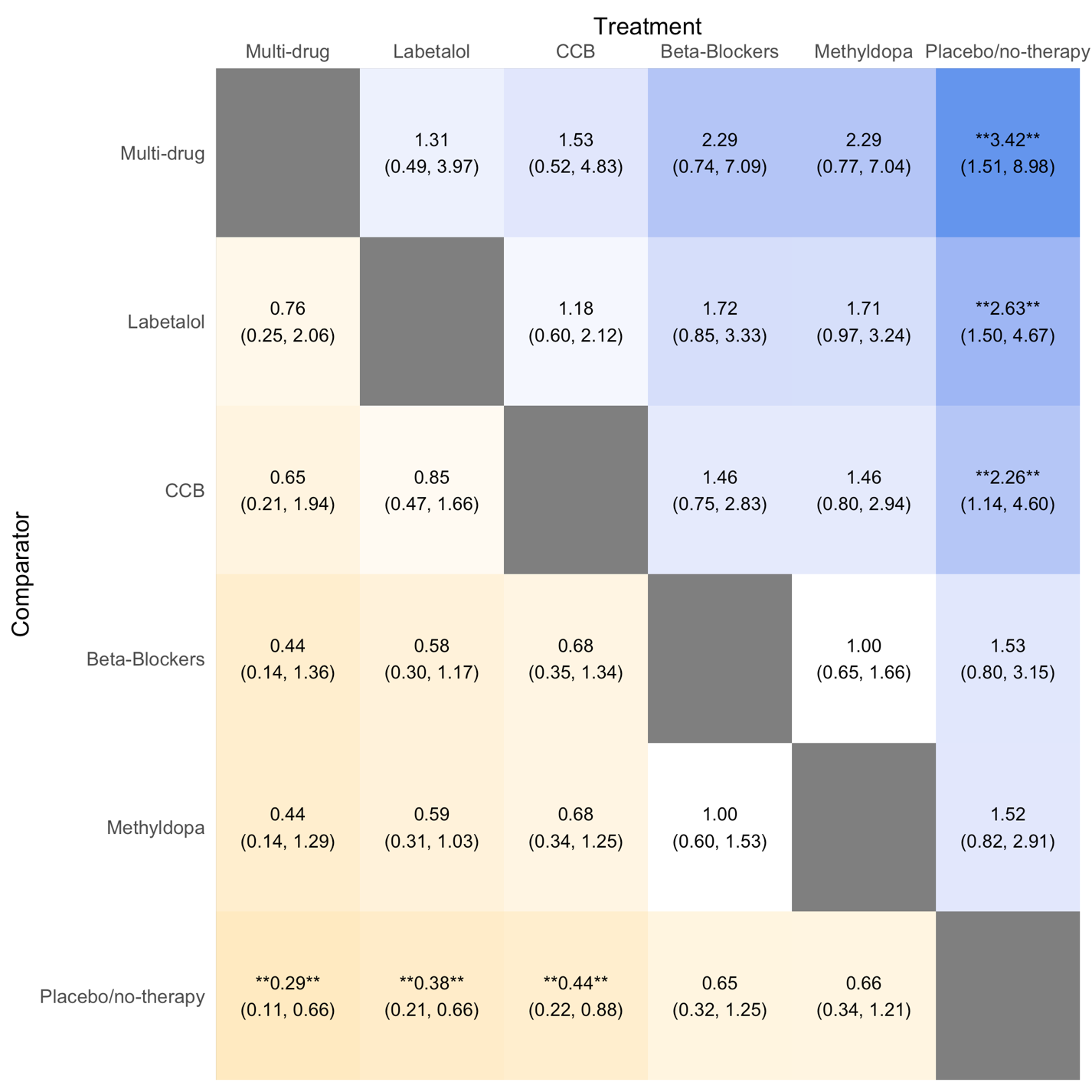
**

**Figure S4B:** Changed/stopped drug due to maternal side effects**
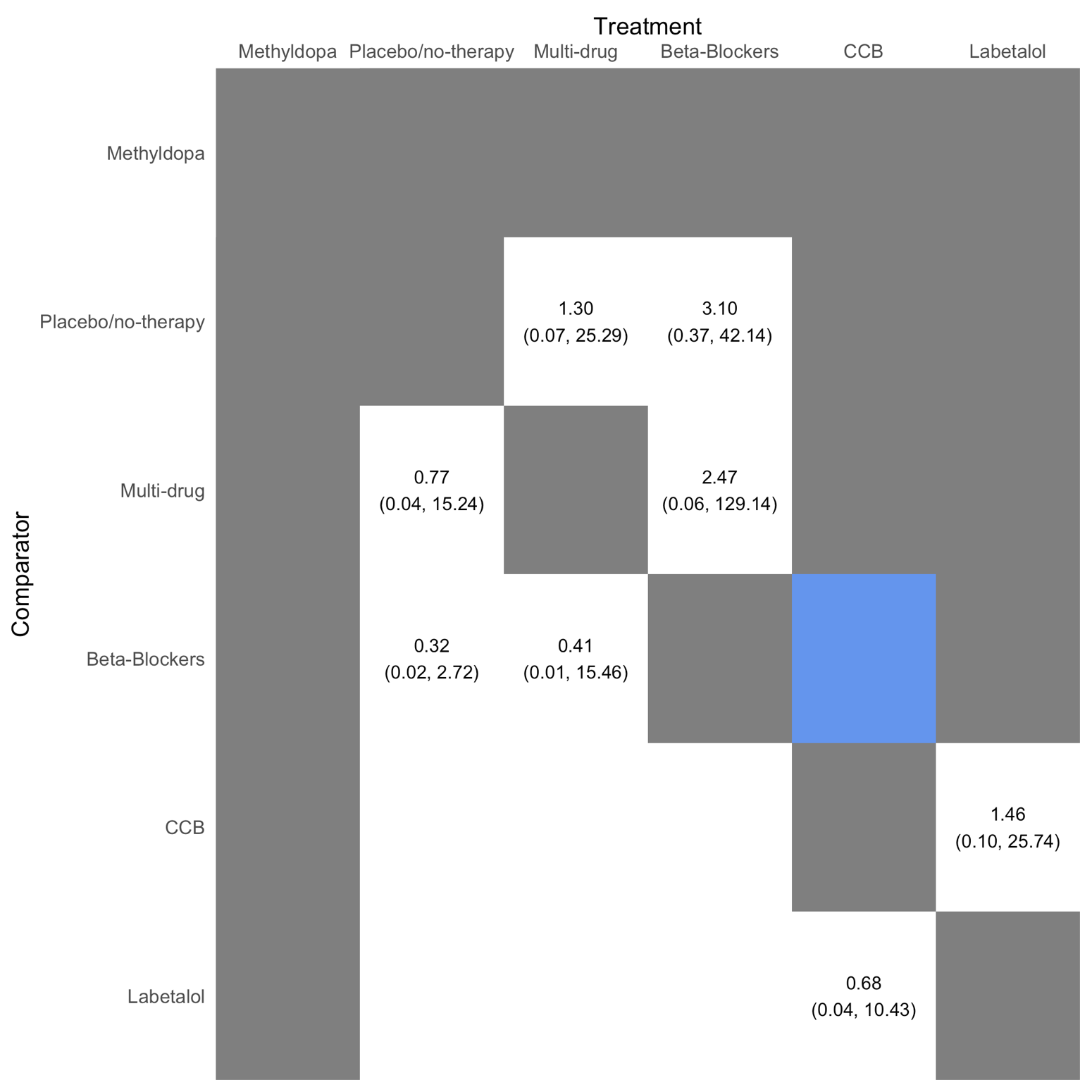
**

**Figure S4C:** Placental abruption **
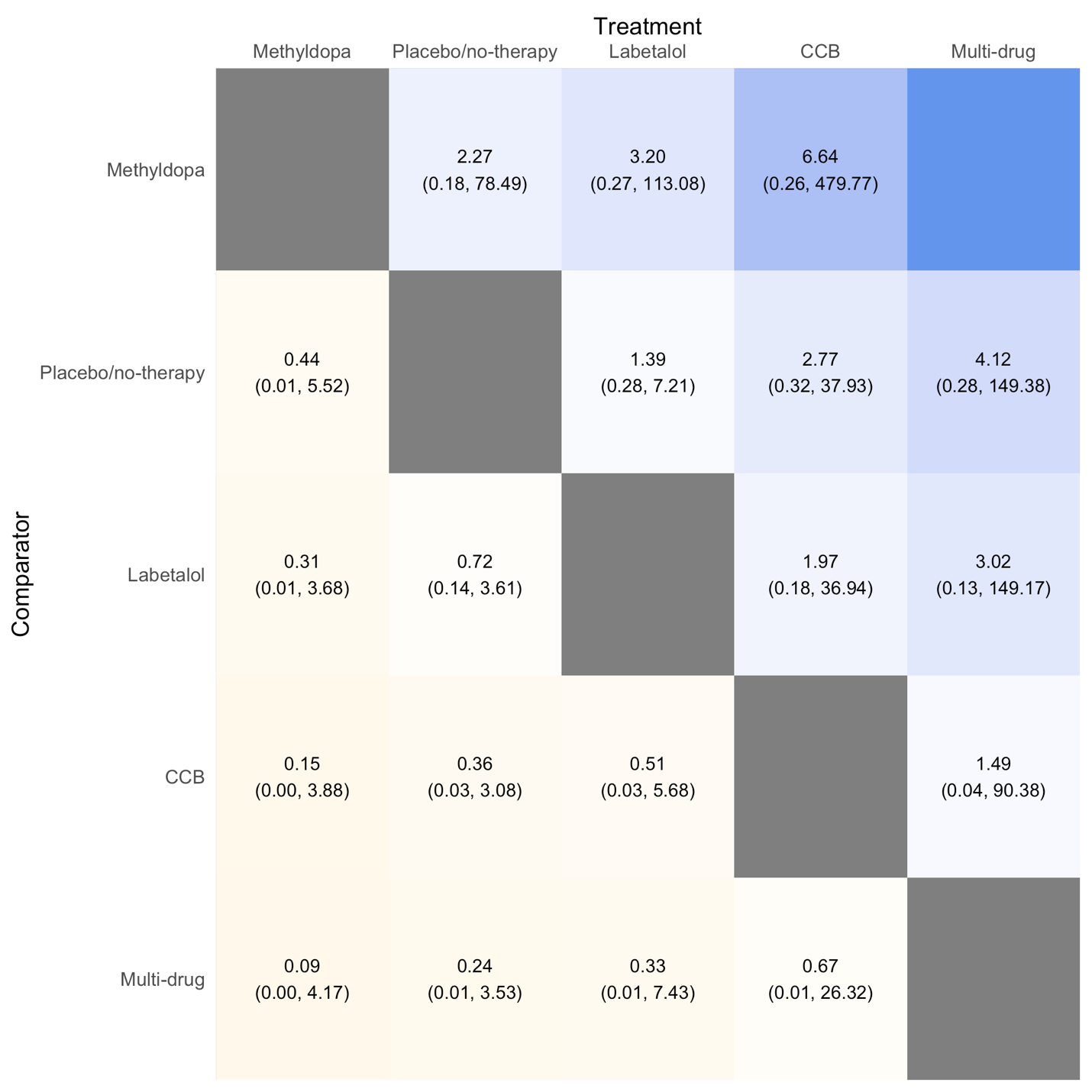
**

**Figure S4D:** Cesarean delivery**
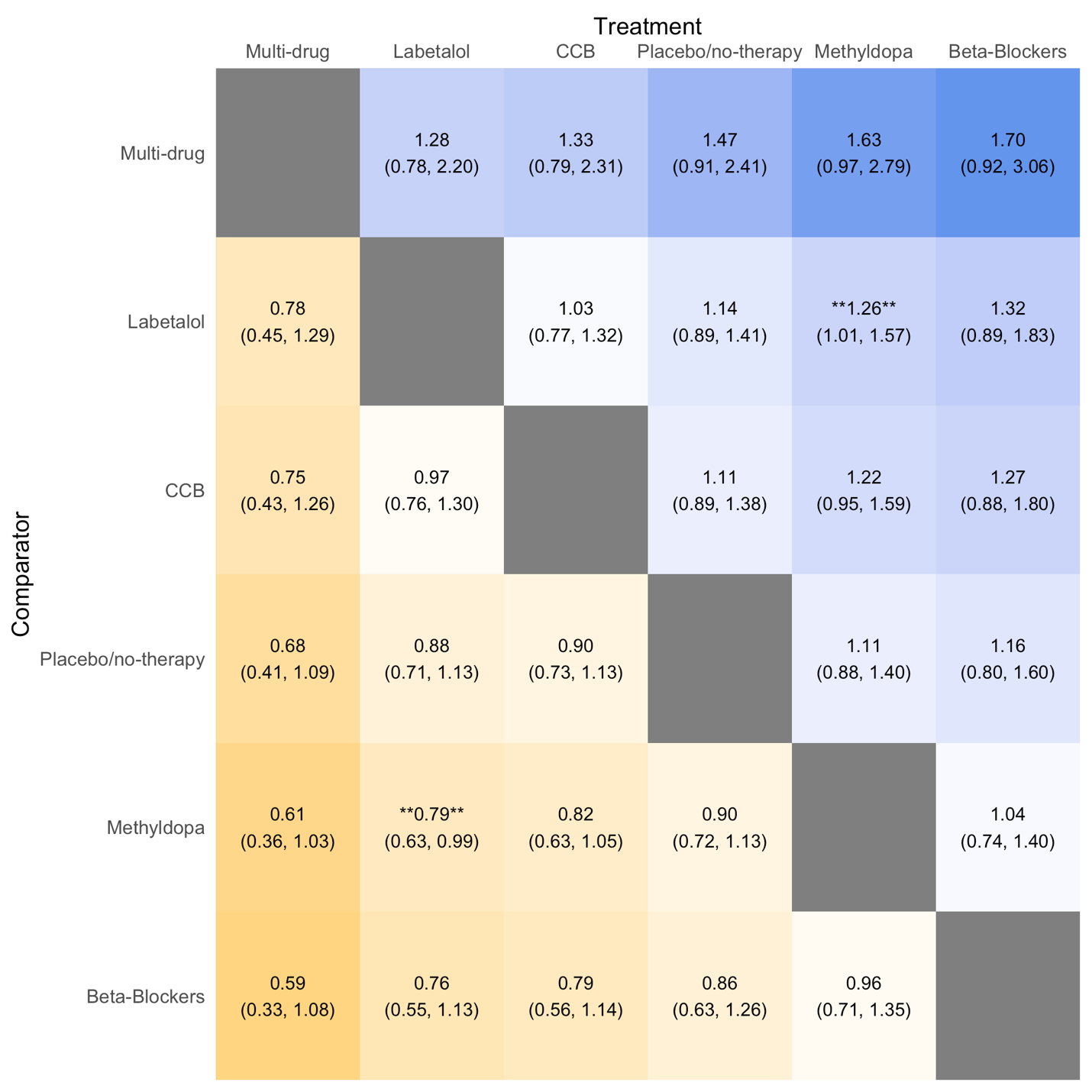
**

**
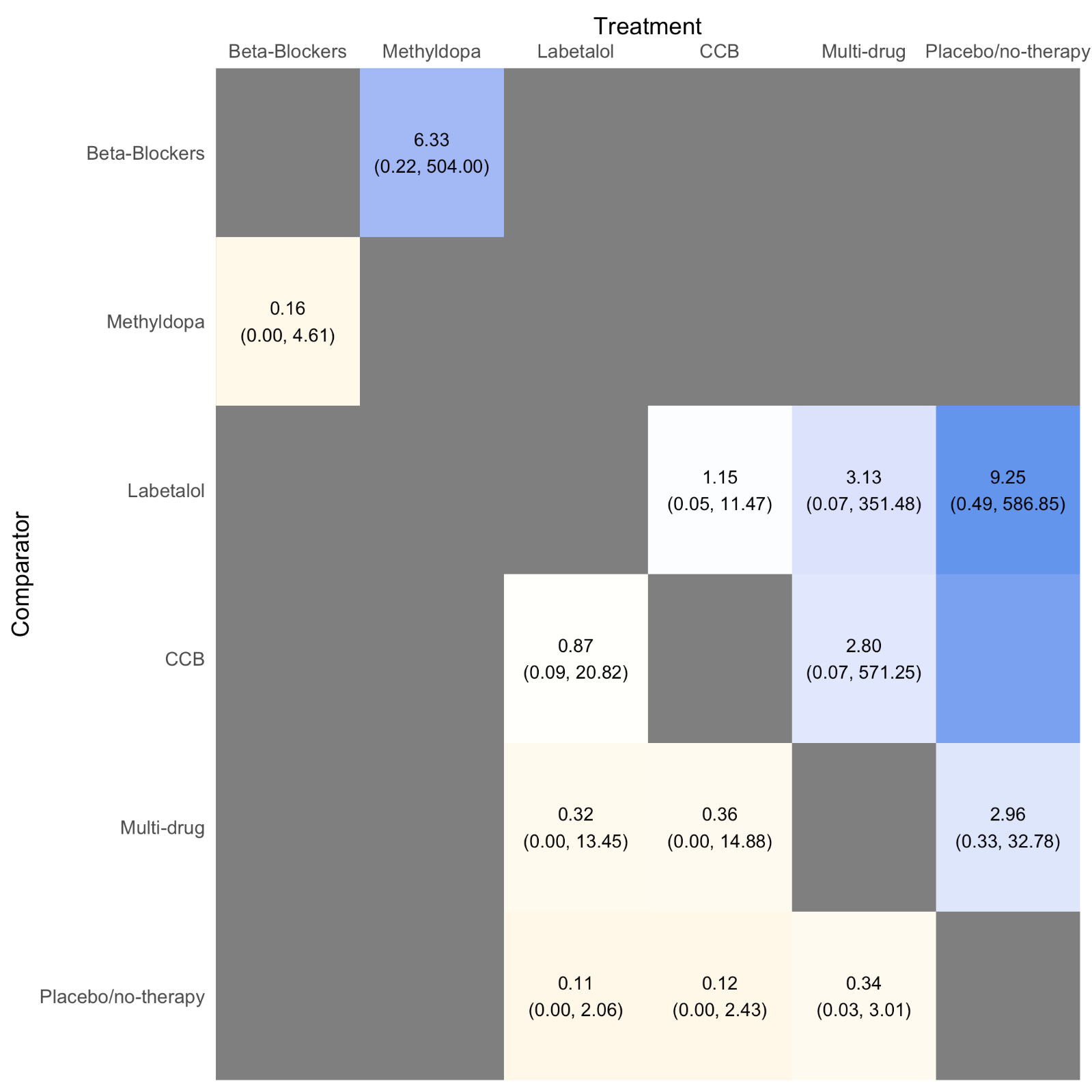
Figure S4E:** Respiratory distress syndrome
