# Supplementary figures and images for: Oral Antihypertensives for Nonsevere Pregnancy Hypertension: Systematic Review, Network Meta- and Trial Sequential Analyses
Source: Hypertension. 2022 Jan 4;79(3):614–28. doi: 10.1161/HYPERTENSIONAHA.121.18415 (PMC8823910; doi:10.1161/HYPERTENSIONAHA.121.18415)

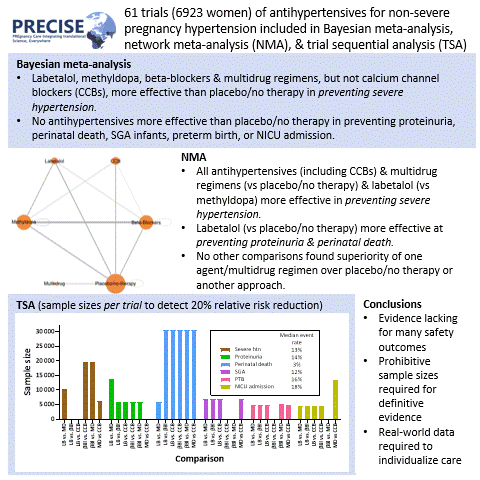

Supplement: Supplementary file 2 [file hyp-79-614-s002.gif]
