# Supplementary material for: Oral Antihypertensives for Nonsevere Pregnancy Hypertension: Systematic Review, Network Meta- and Trial Sequential Analyses
Source: Hypertension. 2022 Jan 4;79(3):614–28. doi: 10.1161/HYPERTENSIONAHA.121.18415 (PMC8823910; doi:10.1161/HYPERTENSIONAHA.121.18415)
Supplement: Supplementary file 3 [file hyp-79-614-s003.pdf]

| | |
|---|---|
| **From:** | Magee, Laura |
| **To:** | Hypertension |
| **Cc:** | Jeffrey Bone |
| **Subject:** | RE: HYPE/2021/18415R1: Request Final Files - style issues |
| **Date:** | Thursday, December 23, 2021 11:36:07 AM |
| **Importance:** | High |

*** **CAUTION:** This email originated from outside of the **American Heart Association**. Do **not** click links or open attachments unless you recognize the sender and know the content is safe. ***

Dear Ms. Gil:

Thank you for this good news. I am in the process of revising the manuscript files and uploading them to the system.

As per your instructions, I'm responding to the references in the **legend of Table 1**. This is an explanation of which trials inform the figures in the table. Is this relevant to ask for permissions. I have, however, corrected the numbering which I had neglected to notice when making the R1 revisions.

I have come up against a couple of problems:

- I had understood that Figure 1 had been sorted between you and Jeff Bone directly. However, now it is too small? I have copied you into the other email string so that hopefully, the three of us can sort this out.
- I read with dismay the supplemental references. Given that this publication is a systematic review, we have over 3500 words in references, which is excessive for a 6000 word maximum. I moved the citations to the supplemental references, which took hours, but I understand now that I cannot do that and they must be cited in the main text first? What is the point of a reference supplement if it is a duplicate? Also, when there are already reference 1, 2, 3, etc, how does one distinguish a reference 1 in the main body from a supplemental reference 1 if there is no 'S'?

Please advise at your earliest convenience.

Many thanks and Merry Christmas.

Kind regards,

Laura

**From:** hypertension@heart.org <hypertension@heart.org>
**Sent:** 16 December 2021 01:24
**To:** Magee, Laura <laura.a.magee@kcl.ac.uk>
**Subject:** HYPE/2021/18415R1: Request Final Files - style issues

PLEASE READ: During this unprecedented and challenging time, the health and safety of you, your

family, and your community is of utmost importance. We appreciate that one aspect of this current situation is the inability to continue research work and that some measures will lead to a significant hindering of research progress. Please know that we are flexible regarding turnaround times for revisions and other tasks during this stressful time and deadlines will be extended as needed. If you are able to, please contact us if you need any extensions or if you experience any challenges around manuscript preparation; we will work with you.

15-Dec-2021

Dr. Laura A. Magee
King's College London
Department of Women and Children's Health
Becket House
1 Lambeth Palace Road
London SE1 7EU
UNITED KINGDOM

MS ID#: HYPE/2021/18415R1
MS TITLE: Oral antihypertensives for non-severe pregnancy hypertension - systematic review, network meta- and trial sequential analyses

Dear Dr. Magee:

I am pleased to inform you that your Original Article is potentially acceptable for publication in Hypertension providing the items below are addressed. Please note that we cannot send your manuscript to production until the items below are addressed completely and in full.

**ACCESS LICENSE RESPONSE REQUIRED:** You previously selected that the Open Access Agreement (CC-BY) authorship forms cover the access licensing REQUIREMENTS OF YOUR FUNDERS for ALL AUTHORS related to this manuscript. Examples include, RUCK, Wellcome Trust, British Heart Foundation, Gates Foundation etc. It is NOT acceptable to change your licensing preference after your manuscript has been accepted, nor after it has gone to production. **Therefore, please respond in email reply, "I confirm selection of Open Access Agreement (CC-BY) MEETS THE NEEDS OF THE FUNDERS OF EVERY AUTHOR and accept responsibility for all costs associated with this choice."**

**PUBLICATION FEES RESPONSE REQUIRED:** Publication fees will apply to your manuscript. All information regarding publication fees can be accessed at https://www.ahajournals.org/hyp/revised-accepted-manuscripts. **After doing so, please also respond in email reply, "I confirm that I have read and am fully aware of the costs associated with publishing my manuscript."**

- Title page - Provide all contact information of the Corresponding Author: email.
- Graphic abstract should be provided as JPG or GIF file AND NOT A JPG FILE. The resolution has to be 300 dpi and the submitted image should be no more than a 15 cm square (15 cm x 15 cm). Do not

include data items; all content should be graphical.
- Text-online supplement and table - please do not highlight the text of the manuscript.
- Legend of table 1 refers to references in the text of the manuscript. Please provide permission to reprint or include a statement that permission is not necessary (and why) by email.
- Figure 1 - increase its resolution from 72 to 300 dpi in a TIFF or EPS file.
- Title page text and online supplement- provide the first name of each author instead of their first initial.
- The font size of the manuscript has to be 12 pt.
- Remove the section Funding after the abstract page and place it after ACKNOWLEDGEMENTS.
- Place Disclosures after Sources of Funding.
- The Abstract must be organized into the following four sections:
1. Background: description of the rationale for the study
2. Methods: brief description of methods
3. Results: presentation of significant results
4. Conclusions: interpretation and significance of the observations, emphasizing new information

- Replace the Novelty and Significance by:
Pathophysiologic Novelty and Relevance:
Please add a Pathophysiologic Novelty and Relevance section with the sub-sections: "What is new," "What is Relevant" and "What are the Pathophysiological Implications" after the reference section.

- Place figure legends after Pathophysiologic Novelty and Relevance.
- Text and online supplement - supplemental references should be cited as 1, 2, 3... etc instead of S1, S2, S3, ... etc.

- Please note that all references must first be mentioned in the manuscript text before mention in the Supplemental Materials alone. To confirm, any references in the Supplemental Material must also be mentioned in the manuscript text. This is for proper indexing of the references and unfortunately, no exceptions in regard to word count will be made.

In addition, all figures must be publication ready prior to final submission. Please refer to https://www.ahajournals.org/pb-assets/migration/Hype_InstructionsforFigureSubmission-1532530896710.pdf for figure requirements.

Authorship changes are not allowed post acceptance. If you make any changes in authorship of your manuscript (additions, deletions, change of order) prior to acceptance, you must complete a Change of Authorship Form and have all authors sign agreeing to the change. If your manuscript is accepted, it will not be possible to publish it until all signatures are in place. The form may be downloaded from https://www.ahajournals.org/pb-assets/migration/COA_Form-1532532203410.pdf.

The files are needed before your manuscript can enter the production cycle. When all listed items are complete please submit online at https://hype-submit.aha-journals.org/cgi-bin/main.plex?el=A1Hm5DBkv1B1CSkT2BS6A9ftdw3IckOQc3E5RGU1A9lf09gZ.

Please contact our editorial office if you have questions about the items requested ( hypertension@heart.org ). We will not be able to submit your manuscript for publication until final materials are received in this editorial office. Also, the date your final materials are received will be published in Hypertension as the acceptance date for your manuscript.

Thanks again for submitting your interesting work to Hypertension. We look forward to receiving future manuscripts from you. With kind regards,

Sincerely,

Renata Gil
Assistant Managing Editor
Hypertension Editorial Office

==========================================
To submit, go to https://hype-submit.aha-journals.org/cgi-bin/main.plex?el=A1Hm5DBkv1B1CSkT2BS6A9ftdw3IckOQc3E5RGU1A9lf09gZ
